# Supplementary material for: Soft corrugated channel with synergistic exclusive discrimination gating for CO2 recognition in gas mixture
Source: Nat Commun. 2023 Jul 15;14:4245. doi: 10.1038/s41467-023-39470-w (PMC10349876; doi:10.1038/s41467-023-39470-w)
Supplement: Supplementary file 1 — Supplementary Information file [file 41467_2023_39470_MOESM1_ESM.pdf]

Supplementary Information

Soft Corrugated Channel with Synergistic Exclusive Discrimination Gating for CO_2 Recognition in Gas Mixture

Gu et al

Supplementary Table 1. List of physical and electronic parameters for the adsorbate molecules.[1-4]

	CO_2	H_2	C_2H_2	Ar	O_2	N_2	CO	CH_4	C_2H_4	C_2H_6
Kinetic diameter (Å)	3.30	2.89	3.30	3.40	3.46	3.64	3.76	3.80	4.1	4.4
Dipole moment (D)	0	0	0	0	0	0	0.117	0	0	0
Quadrupole moment 10^{40} θ (cm^2)	13.4	2.21	20.4	0	1.3	4.7	8.3	0	5	2.17
Polarizability ($Å^3$)	2.65	0.82	3.59	1.66	1.60	1.76	1.95	2.60	4.25	4.45

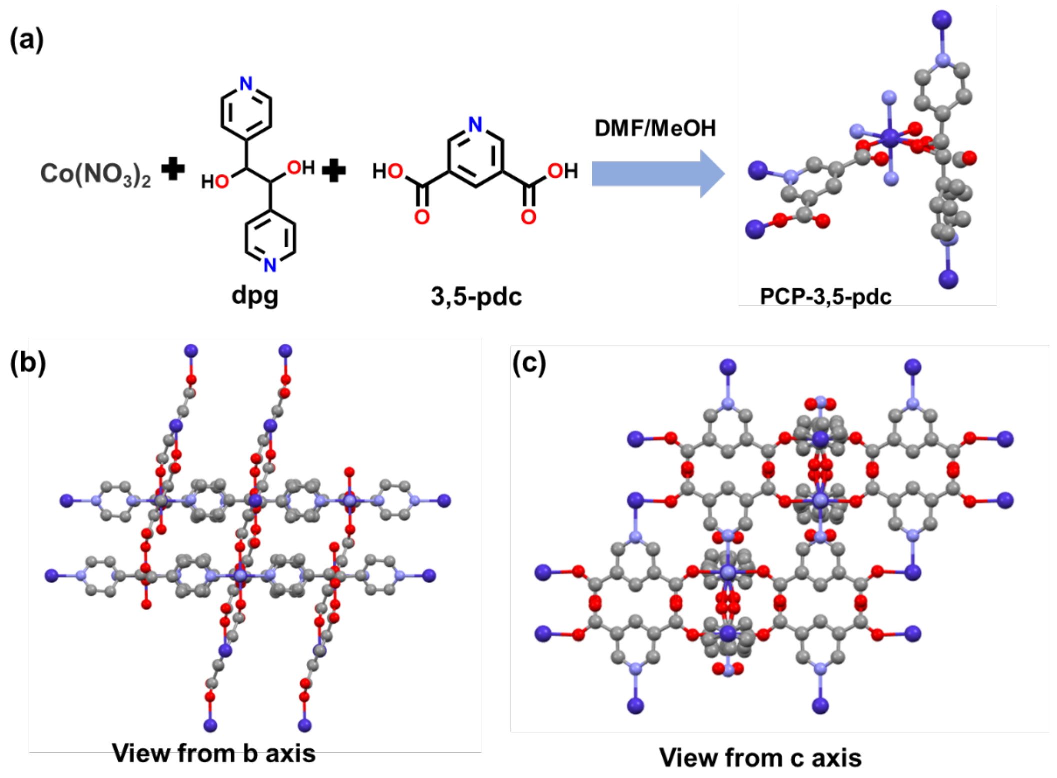

Supplementary Figure 1. (a) Scheme of synthesis of PCP-3,5-pdc. (b-c) The structure of the as-synthesised of PCP-3,5-pdc. Purple, Red, blue and grey colours in the PCP frameworks represent Co, O, N and C, respectively. The hydrogen atoms and guest solvent molecules are omitted for clarity.

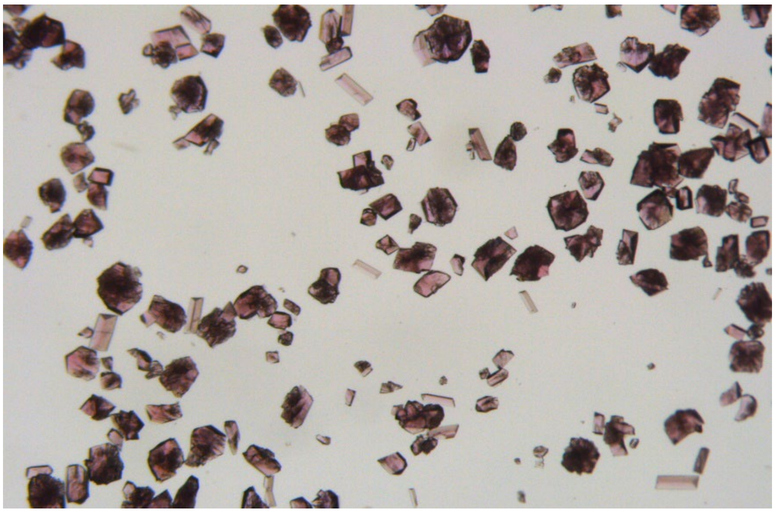

Supplementary Figure 2. Photo of as-synthesised single crystals of PCP-3,5-pdc under microscope.

Supplementary Table 2. Crystallographic data and structural refinement summary for as-synthesised PCP-3,5-pdc.

	As-synthesised PCP-3,5-pdc
Chemical formula	C19H13CoN3O6
Formula weight	438.25
Crystal system	monoclinic
Space group	I 2/a
a/Å	11.0422 (3)
b/Å	15.6259 (6)
c/Å	13.6251 (5)
α/Å	90
β/°	101.321 (3)
γ/°	90
Cell volume / $Å^3$	2305.19(14)
Z	4
Wavelength/Å	0.71073
R_{int}	0.0561
$\mu(cm^{-1})$	0.779
D_{calcd}/ Mg m^{-3}	1.263
F(000)	892
Goodness of fit	1.047
Temperature (K)	100
Reflections collected	3437
Independent reflections	3020
R_1 ($I > 2.00\sigma(I)$, all data)	0.0656, 0.0582
wR_2 ($I > 2.00\sigma(I)$, all data)	0.1610, 0.1558

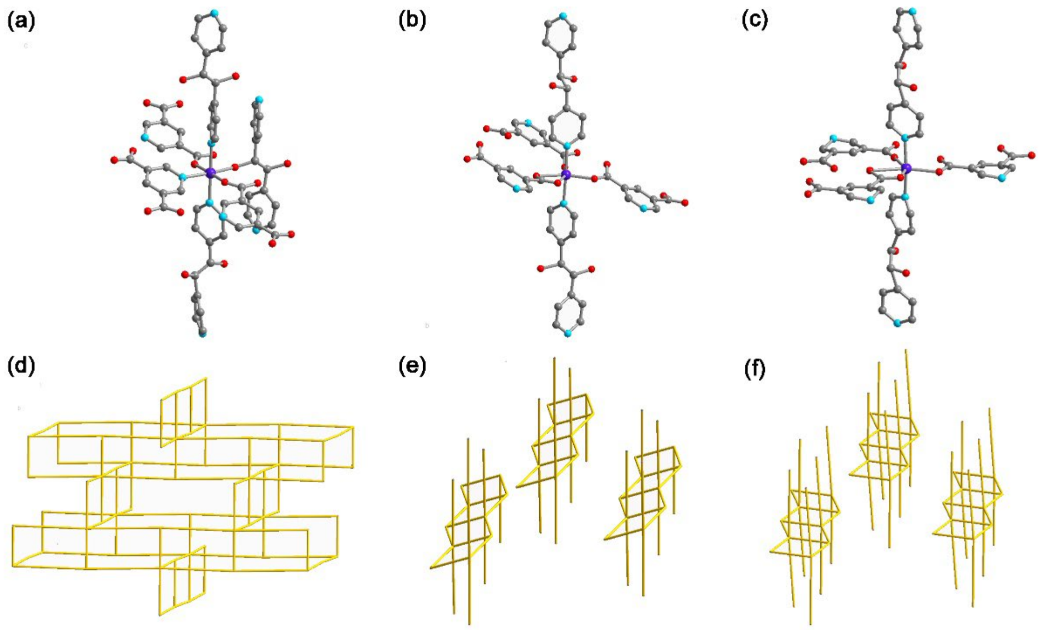

Supplementary Figure 3. (a-c) The coordination environment of Co(II) in phase α, β and γ of PCP-3,5-pdc. (d-f) The simplified topological structures of phase α, β and γ of PCP-3,5-pdc. In the as-synthesised PCP-3,5-pdc, each Co(II) is coordinated to three 3,5-pdc and three dpg ligands. In the activated and CO_2 loaded PCP-3,5-pdc, each Co(II) is coordinated to three 3,5-pdc and two dpg ligands.

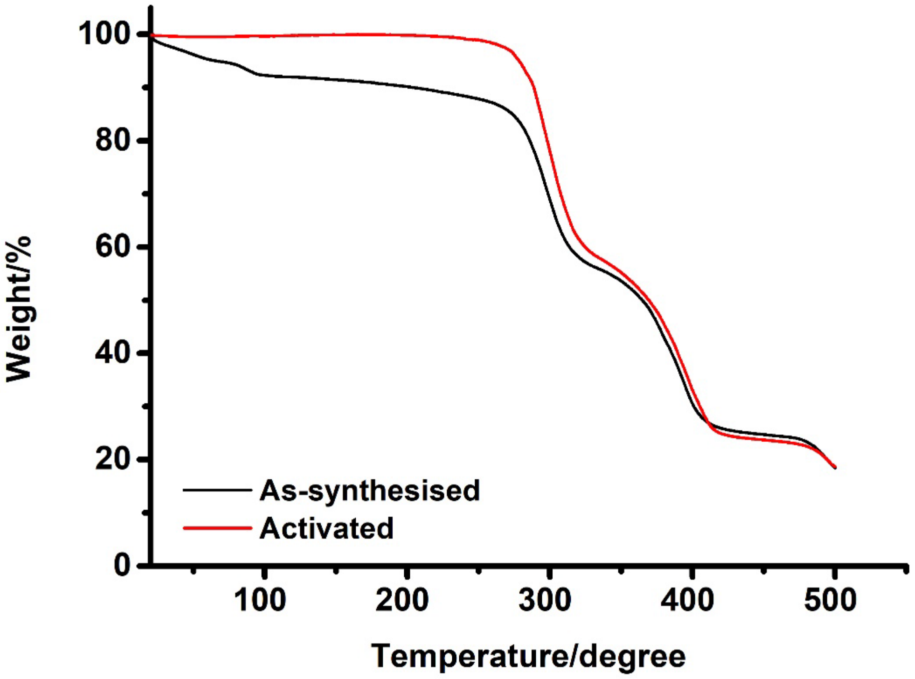

Supplementary Figure 4. TGA analysis of as-synthesised and activated PCP-3,5-pdc. The coordination framework of PCP-3,5-pdc is thermally stable up to 270 ºC.

Supplementary Table 3. Crystallographic data and structural refinement summary for activated PCP-3,5-pdc.

	Activated PCP-3,5-pdc
Chemical formula	C19H13CoN3O6
Formula weight	438.25
Crystal system	triclinic
Space group	$P\bar{1}$
a/Å	10.0698(9)
b/Å	10.2555(9)
c/Å	10.5581(10)
α/Å	81.633(8)
β/°	62.416(9)
γ/°	78.554(7)
Cell volume / $Å^3$	945.38(17)
Z	2
Wavelength/Å	0.71073
R_{int}	0.0782
$\mu(cm^{-1})$	0.950
D_{calcd}/ Mg m^{-3}	1.540
$F(000)$	446
Goodness of fit	1.100
Temperature (K)	100
Reflections collected	4935
Independent reflections	2600
R_1 ($I > 2.00\sigma(I)$, all data)	0.1694, 0.0864
wR_2 ($I > 2.00\sigma(I)$, all data)	0.2291, 0.1876

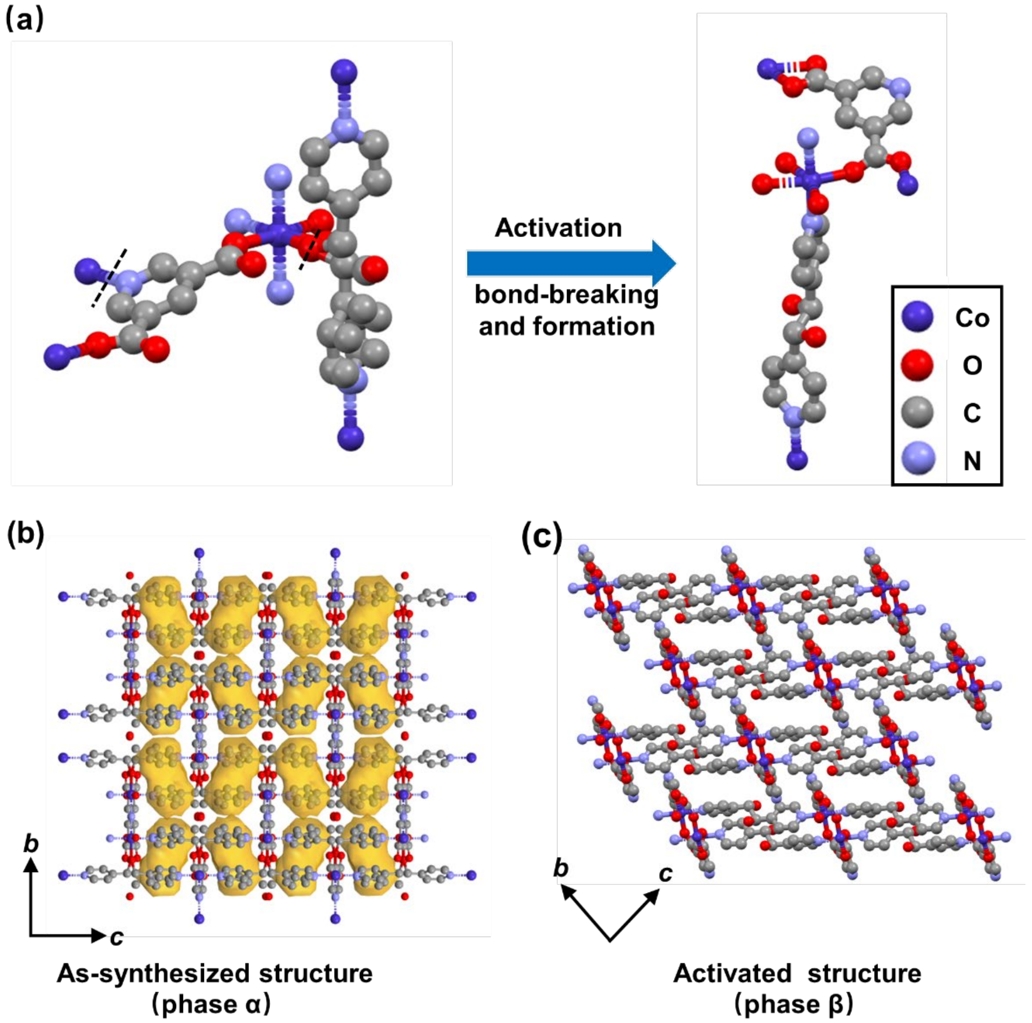

Supplementary Figure 5. (a) Coordination environment of Co in as-synthesised and activated PCP-3,5-pdc. (Dotted lines indicate the bond-breaking part during activation process). (b) Overall framework of the as-synthesised structure of PCP-3,5-pdc (phase α). The isolated voids within the framework are highlighted in yellow (Probe radius: 1.2 Å). (c) Overall framework of the activated structure of PCP-3,5-pdc (phase β). The hydrogen atoms and guest solvent molecules are omitted for clarity.

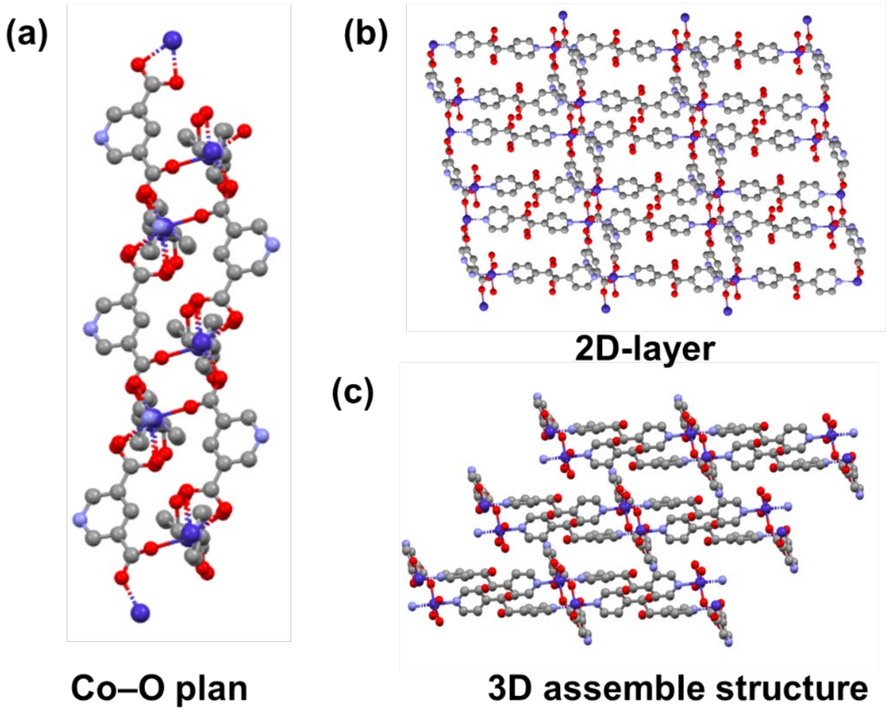

Supplementary Figure 6. The structure of activated PCP-3,5-pdc (phase β). Purple, Red, blue and grey colours in the PCP frameworks represent Co, O, N and C, respectively. The hydrogen atoms are omitted for clarity.

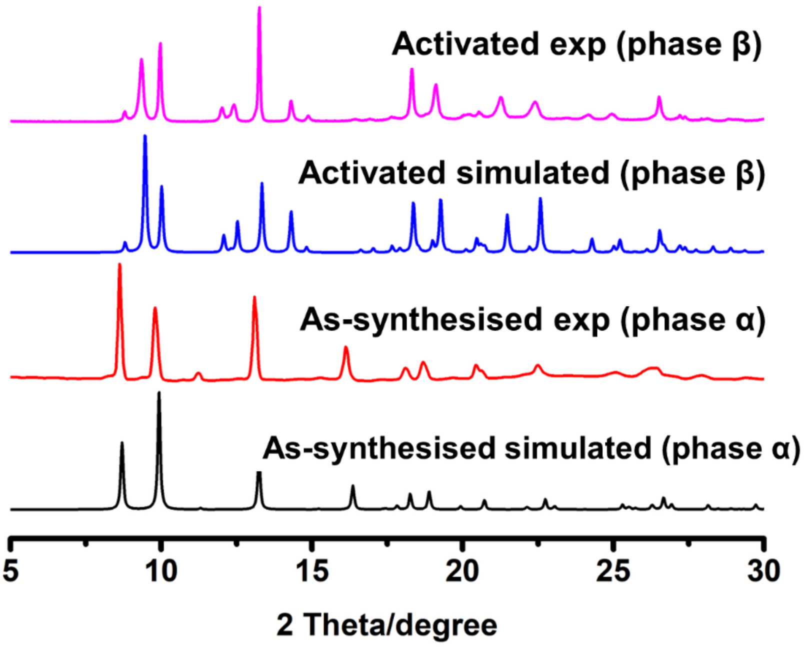

Supplementary Figure 7. XRD patterns of as-synthesised and activated PCP-3,5-pdc.

Supplementary Table 4. The calculated void ratio in phase α, β and γ of PCP-3,5-pdc (probe radius: 1.2 Å)

	Phase α	Phase β	Phase γ
Void ratio	15.4%	3.8%	19.8%

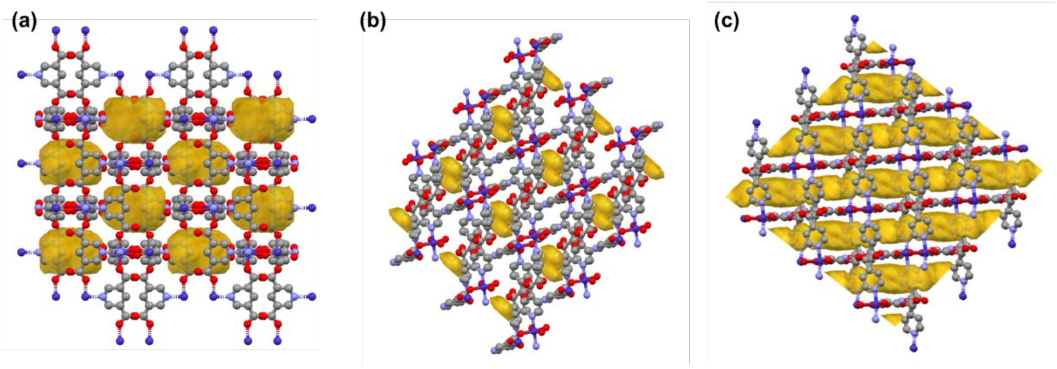

Supplementary Figure 8. The voids in phase α (a), β (b) and γ (c) of PCP-3,5-pdc (Highlighted in yellow, probe radius: 1.2 Å)

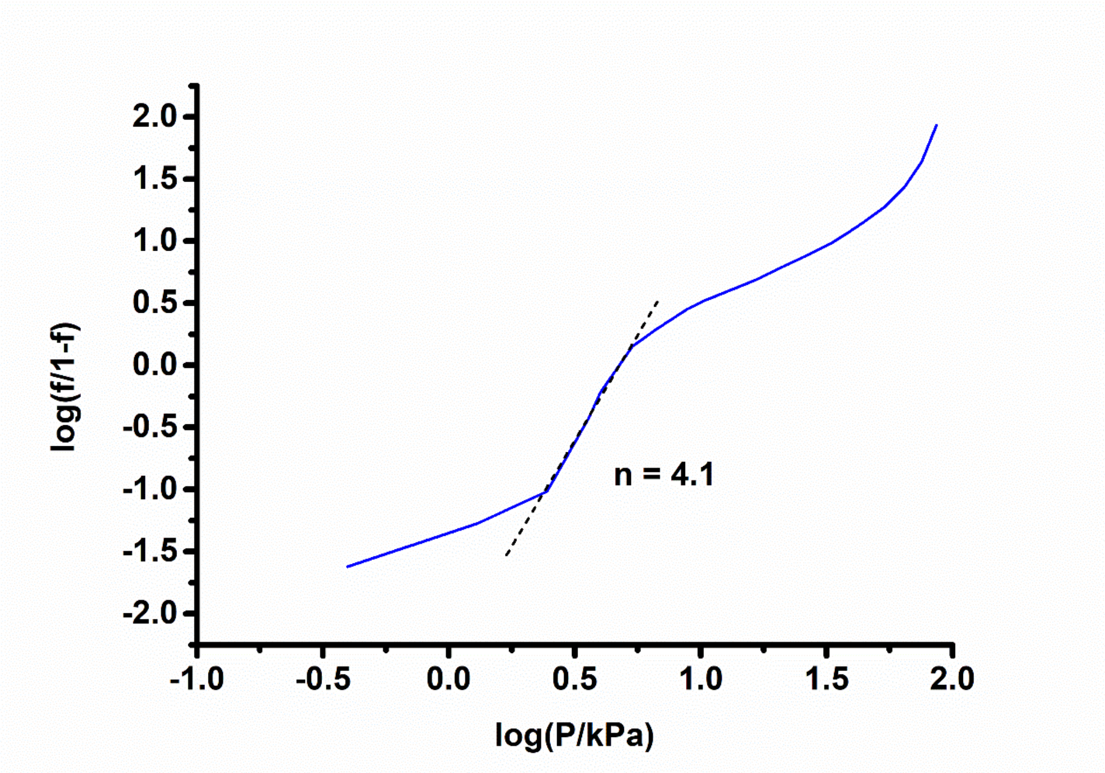

Supplementary Figure 9. Hill analysis of CO_2 adsorption isotherm measured at 195 K in PCP-3,5-pdc.

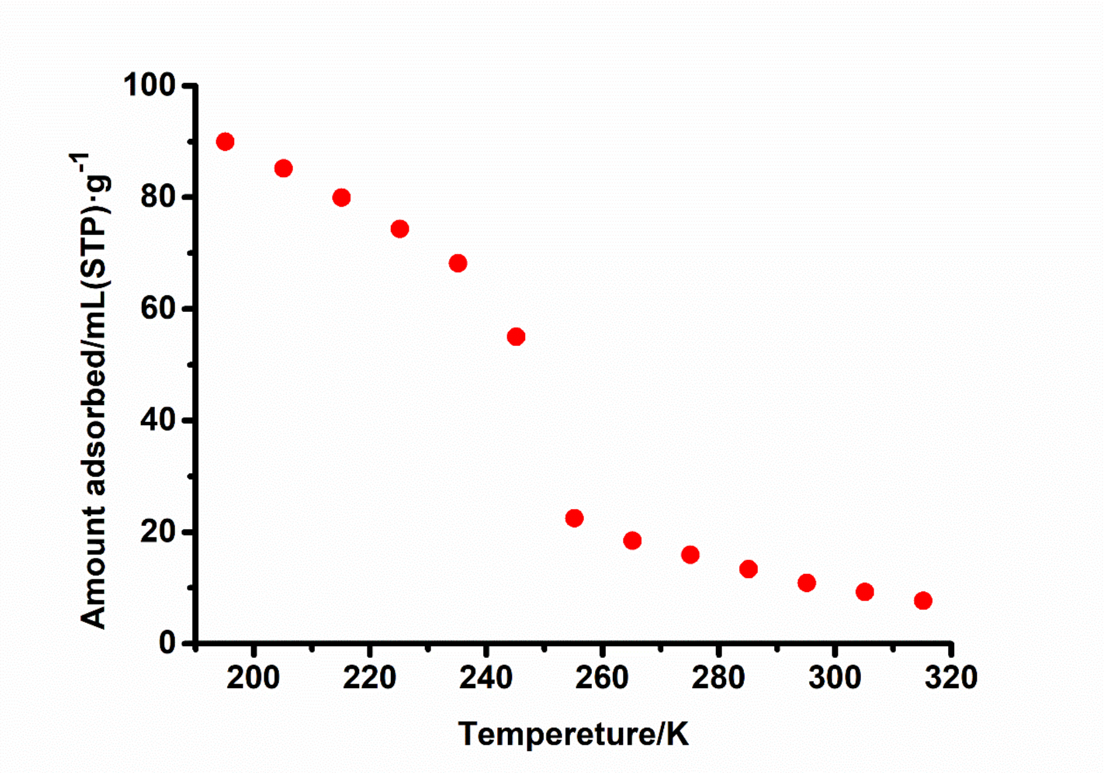

Supplementary Figure 10. Gas adsorption isobar curves of CO_2. The isobar measurements were conducted from low temperature to high temperature.

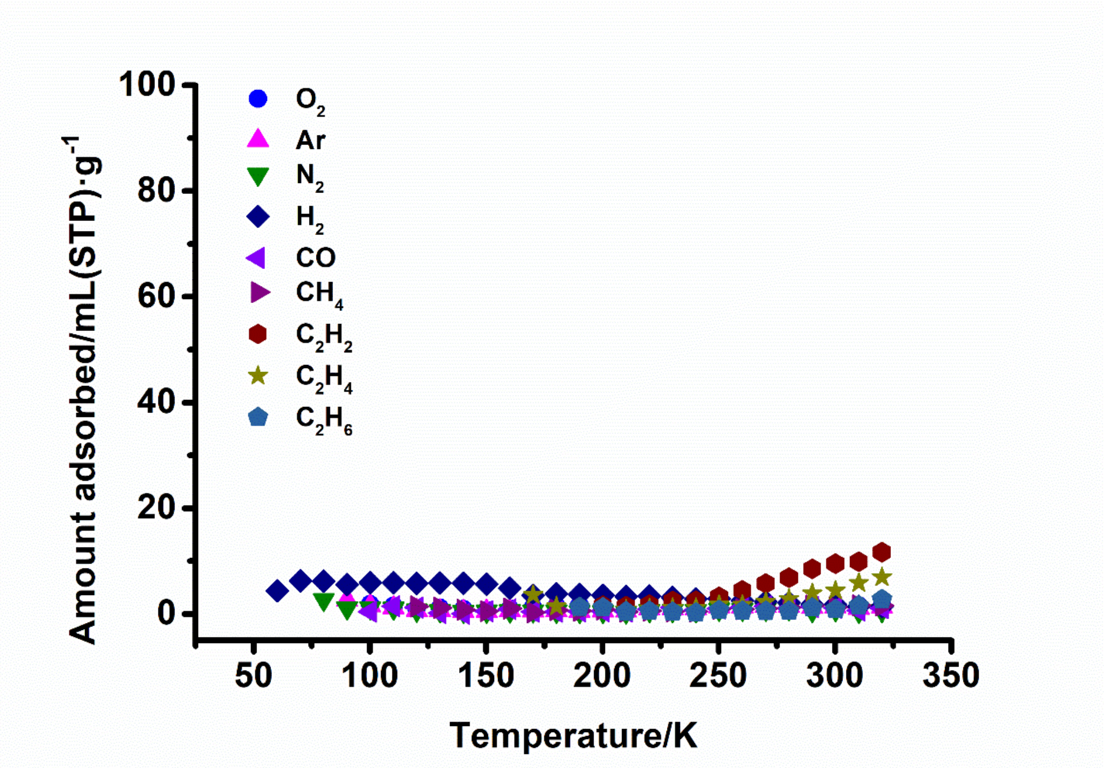

Supplementary Figure 11. Gas adsorption isobar measurements of N_2, CO, C_2H_2, O_2, H_2, Ar, CH_4, C_2H_4, C_2H_6. The isobar measurements were conducted from low temperature to high temperature. Only H_2 sorption was tested from 60 K due to the measurement limitation.

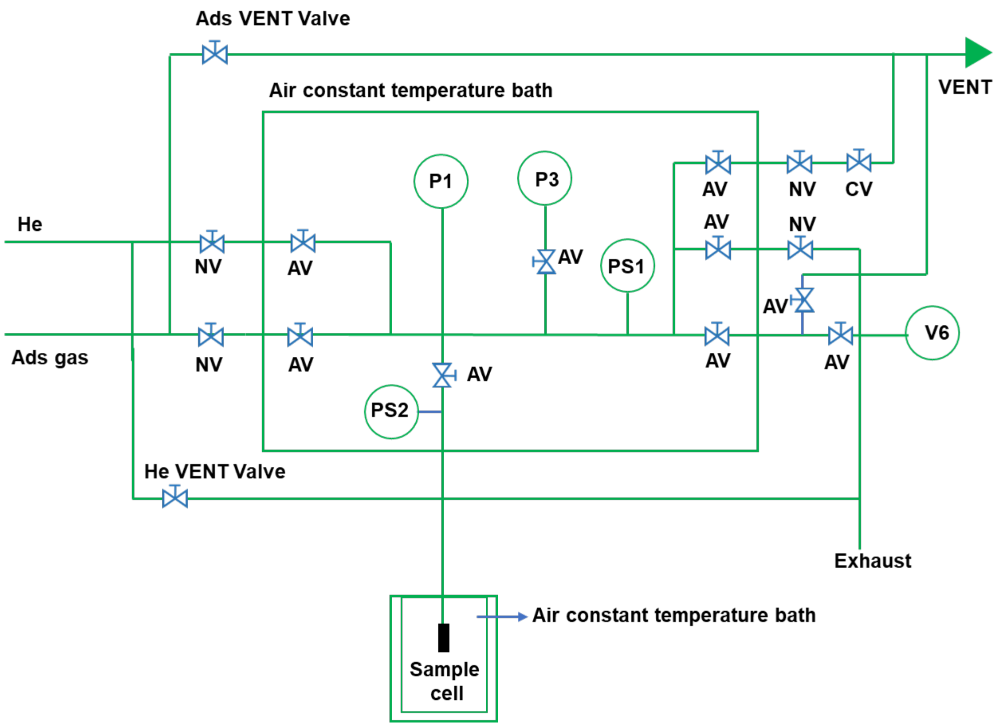

Supplementary Figure 12. Measurement set-up of the used BELSORP HP instrument for high-pressure sorption experiments (AV = air-operated valve, NV = needle valve, CV = check valve, P / PS= pressure sensor). The flow diagram is shown in green color.

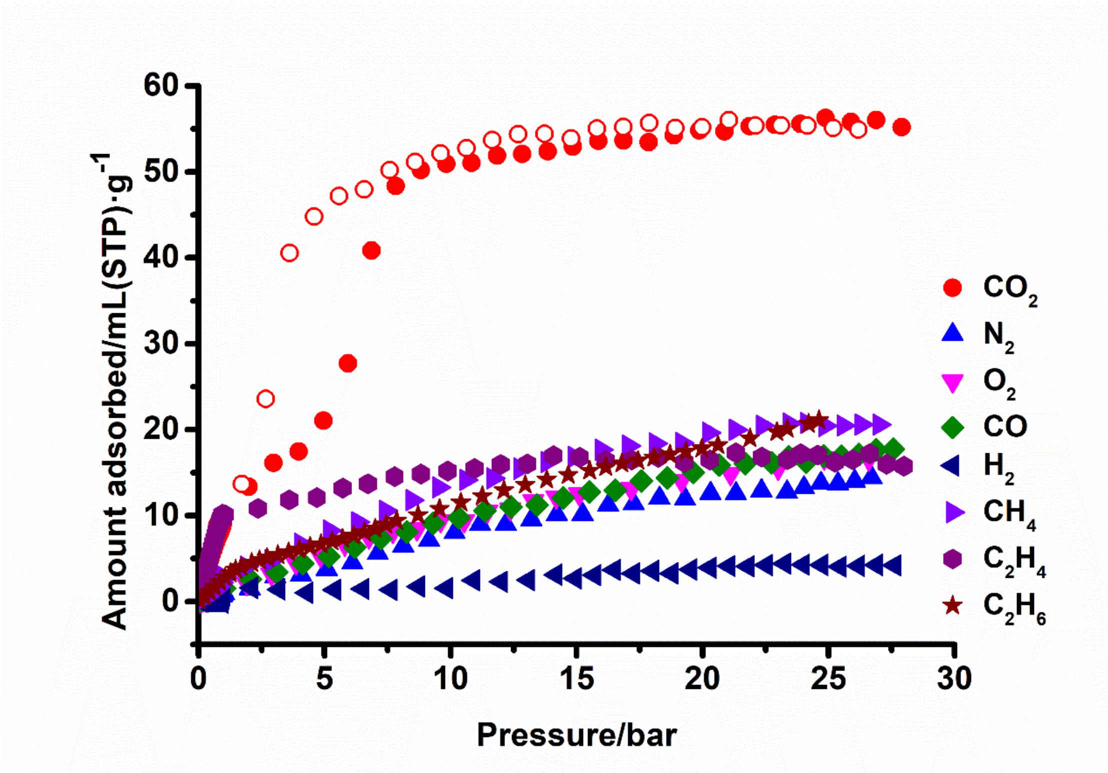

Supplementary Figure 13. High-pressure gas sorption of N_2, CO, C_2H_2, O_2, H_2, Ar, CH_4, C_2H_4, C_2H_6 at 298 K.

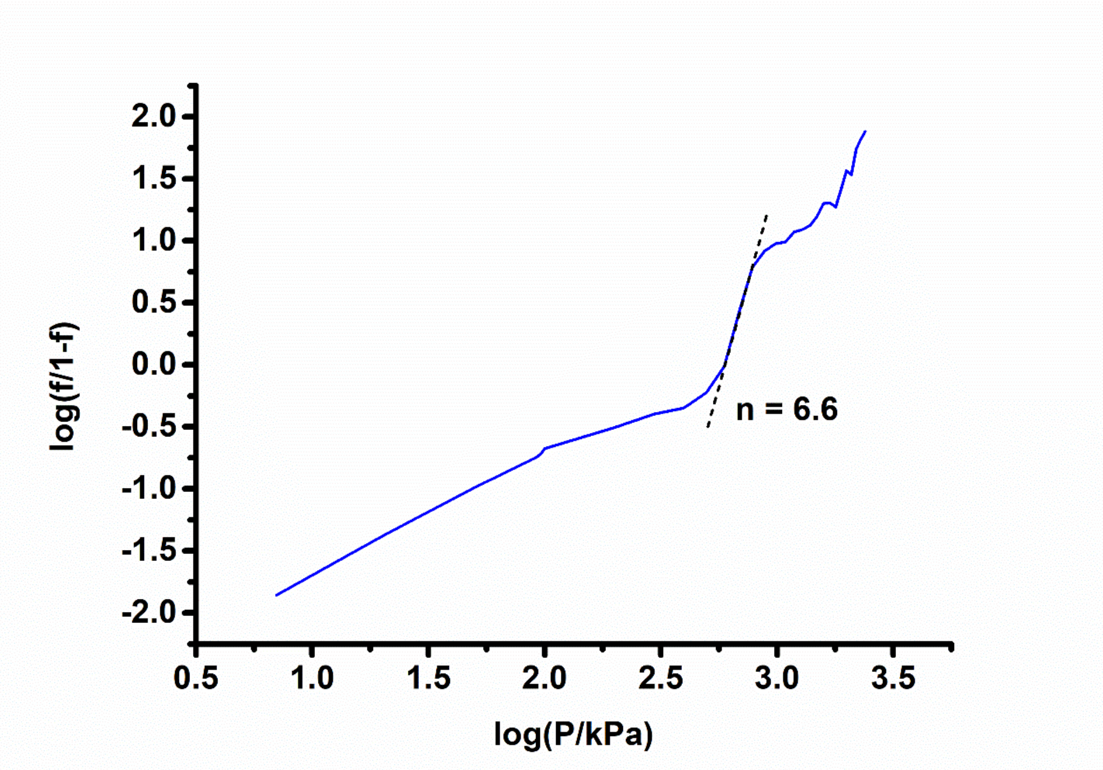

Supplementary Figure 14. Hill analysis of high-pressure CO_2 adsorption isotherm measured at 298 K in PCP-3,5-pdc.

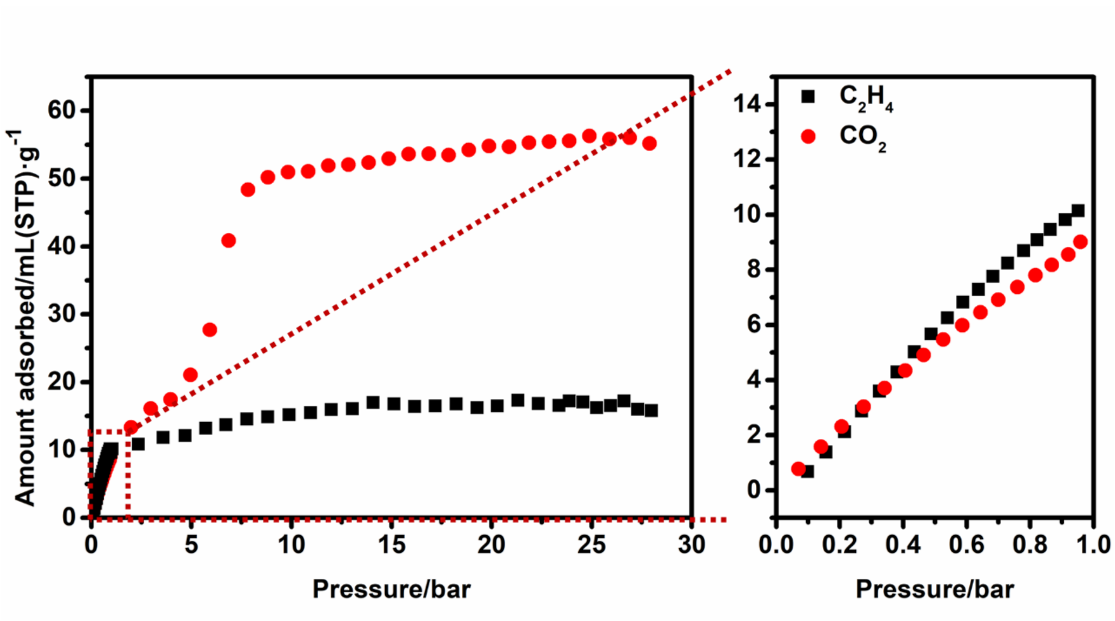

Supplementary Figure 15. High-pressure gas sorption of C_2H_4 and CO_2 at 298 K. The sorption patterns at low pressure region are enlarged in left figure.

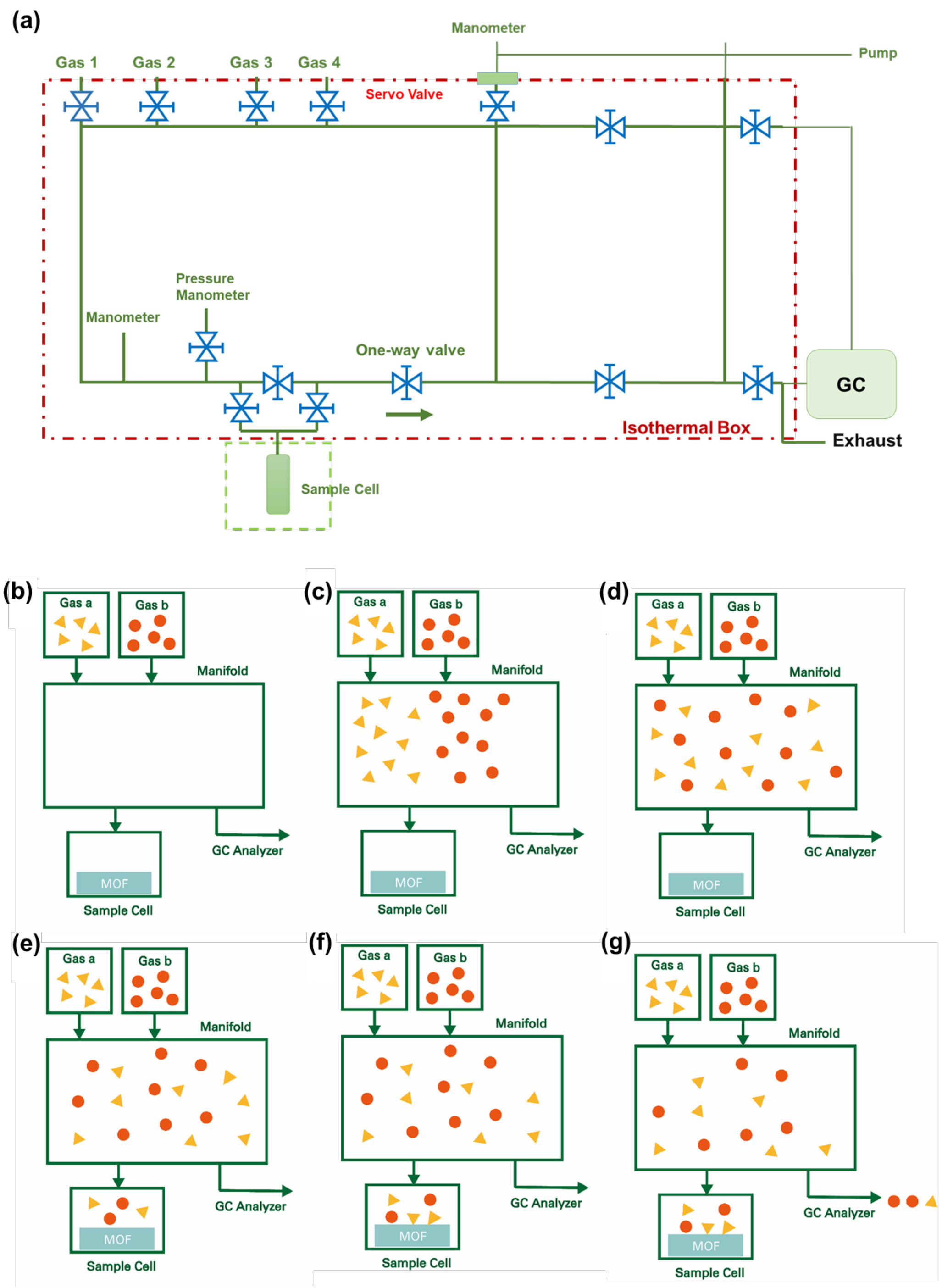

Supplementary Figure 16. (a) Measurement set-up of the used Belsorp VC instrument for high-pressure co-sorption experiments. (b-g) Description of the measurement principle step-by-step[5].

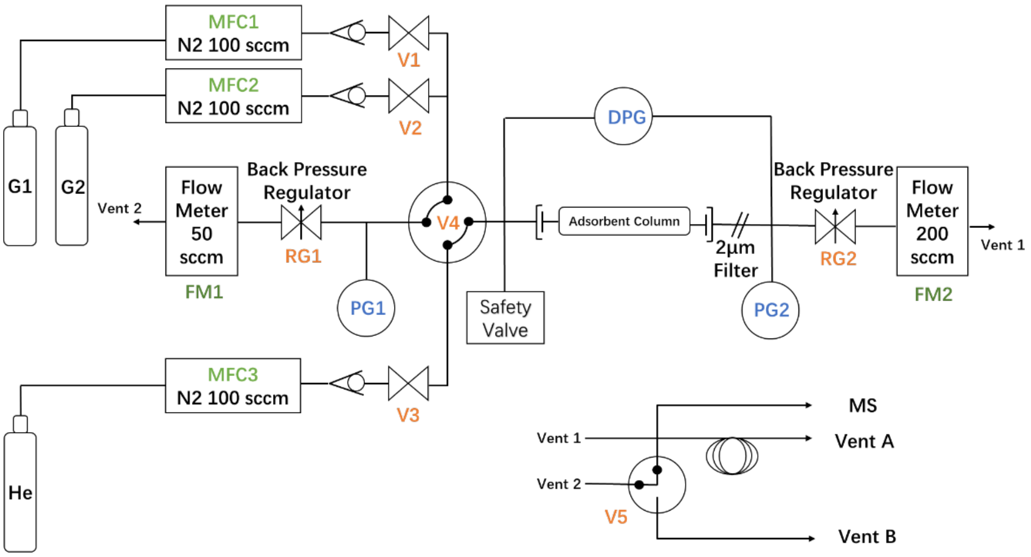

Supplementary Figure 17. The high-pressure breakthrough system used in this study.

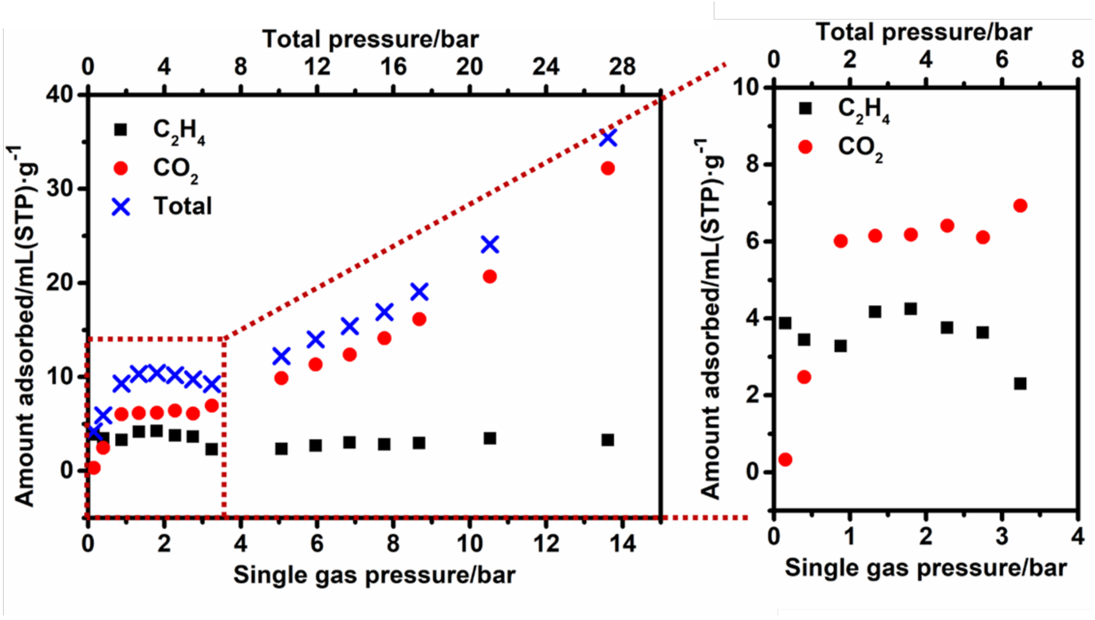

Supplementary Figure 18. Volumetric high-pressure co-adsorption of a binary mixture C_2H_4/CO_2 (50.0/50.0 V/V) at 298 K of PCP-3,5-pdc. The sorption patterns at low pressure region are enlarged in left figure.

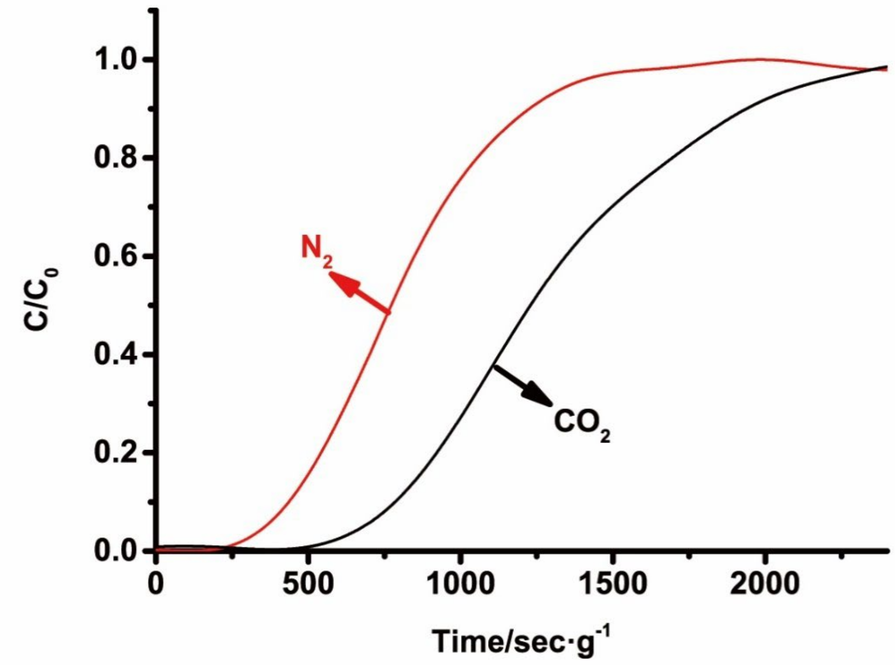

Supplementary Figure 19. Experimental breakthrough curve of PCP-3,5-pdc at a flow rate of 6 mL/min for an equimolar gaseous mixture of N_2 and CO_2 (v/v, 50/50) at room temperature (Total pressure 20 bar).

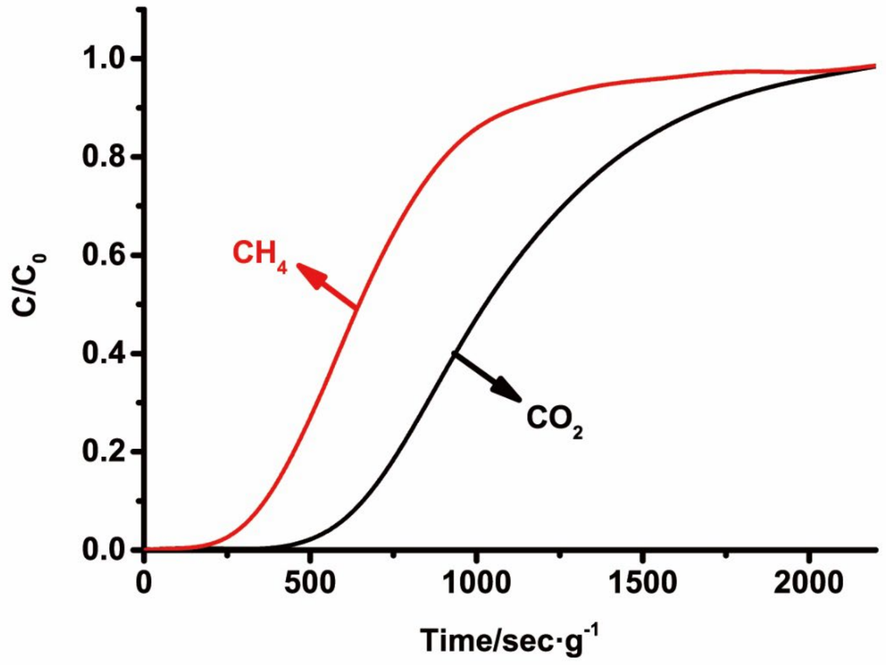

Supplementary Figure 20. Experimental breakthrough curve of PCP-3,5-pdc at a flow rate of 6 mL/min for an equimolar gaseous mixture of CH_4 and CO_2 (v/v, 50/50) at room temperature (Total pressure 20 bar).

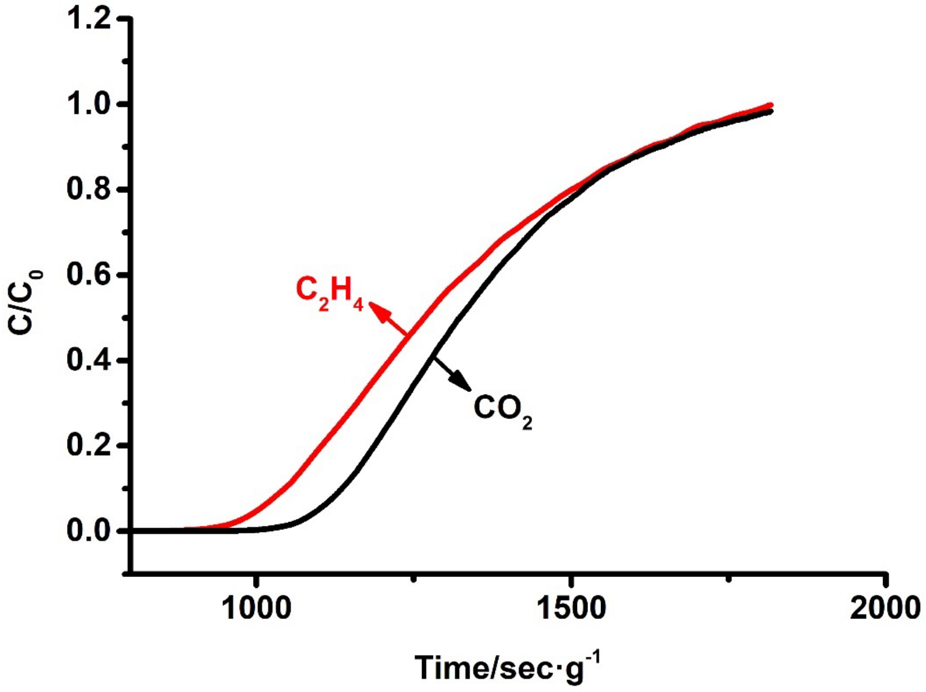

Supplementary Figure 21. Experimental breakthrough curve of PCP-3,5-pdc at a flow rate of 6 mL/min for an equimolar gaseous mixture of C_2H_4 and CO_2 (v/v, 50/50) at room temperature (Total pressure 20 bar).

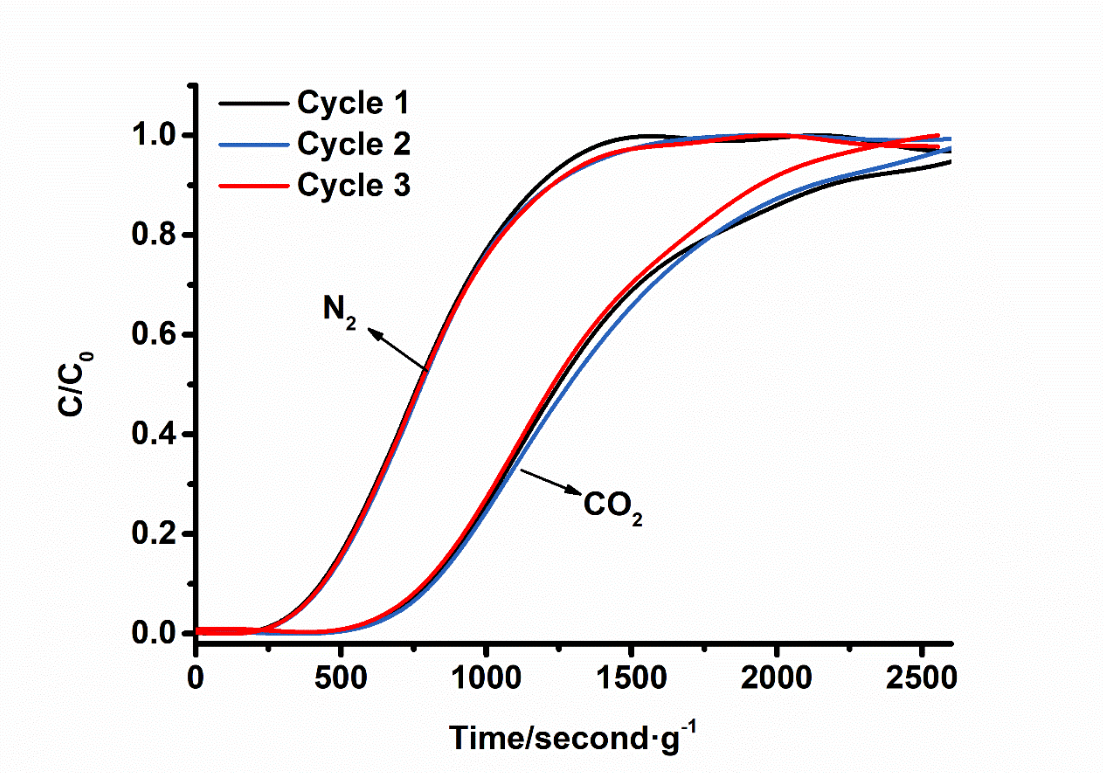

Supplementary Figure 22. Three cycles of experimental breakthrough curve of PCP-3,5-pdc under flow at 6 mL/minute for an equimolar gas mixture of N_2 and CO_2 (50/50, v/v) at room temperature (Total pressure: 20 bar). Between each cycle, the PCPs were regenerated by in situ vacuum without heating.

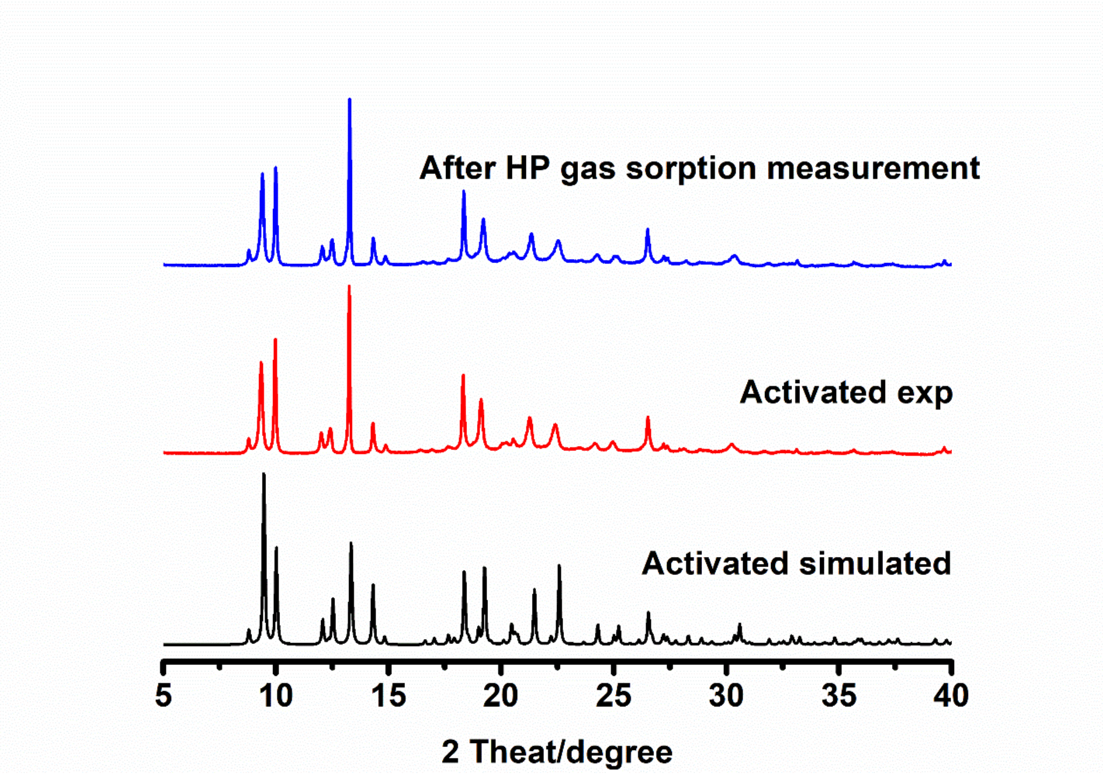

Supplementary Figure 23. PXRD patterns of PCP-3,5-pdc before and after high-pressure (HP) sorption measurement.

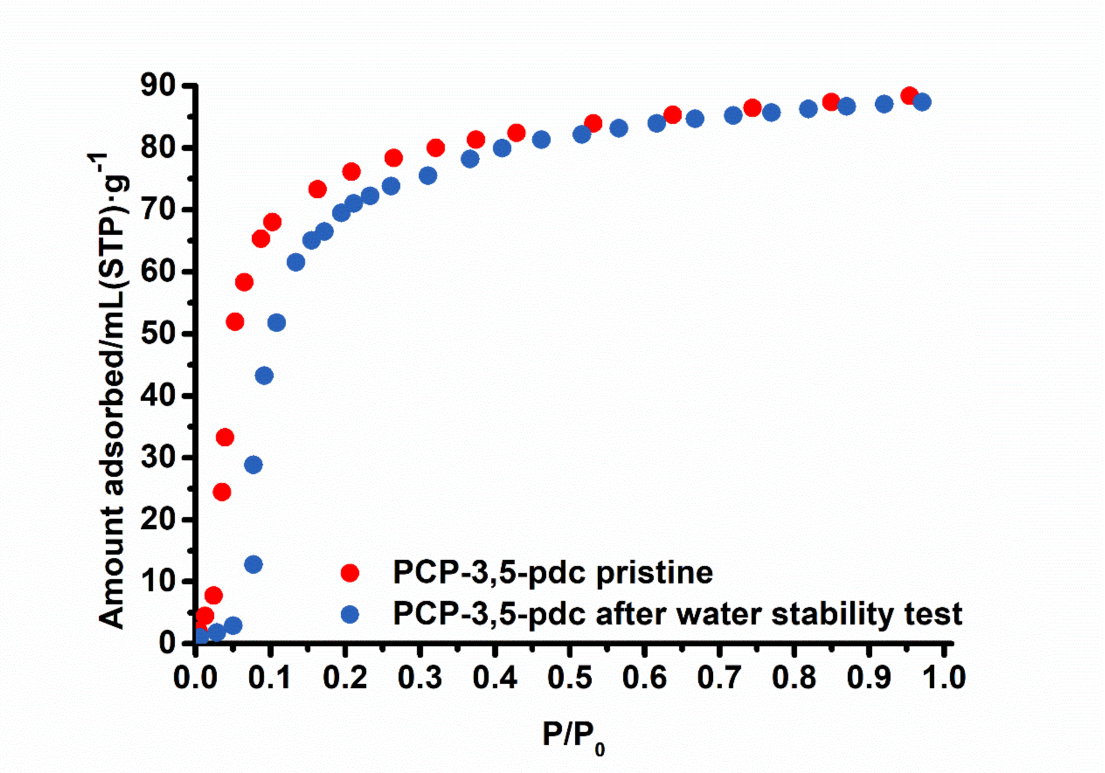

Supplementary Figure 24. CO_2 adsorption isotherms at 195 K of PCP-3,5-pdc before and after water stability test.

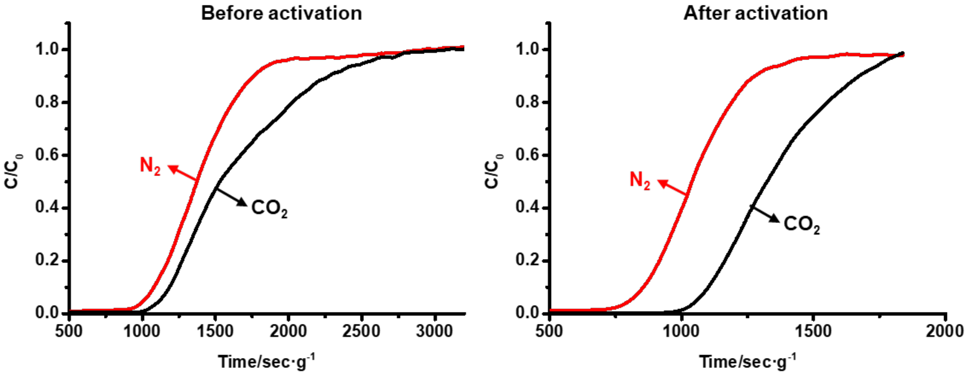

Supplementary Figure 25. Experimental breakthrough curve of PCP-3,5-pdc before and after activation (at 373 K under vacuum for 2 hours), after exposure to humidity for more than one week at a flow rate of 6 mL/min for an equimolar gaseous mixture of N_2 and CO_2 (v/v, 50/50) at room temperature (Total pressure 20 bar).

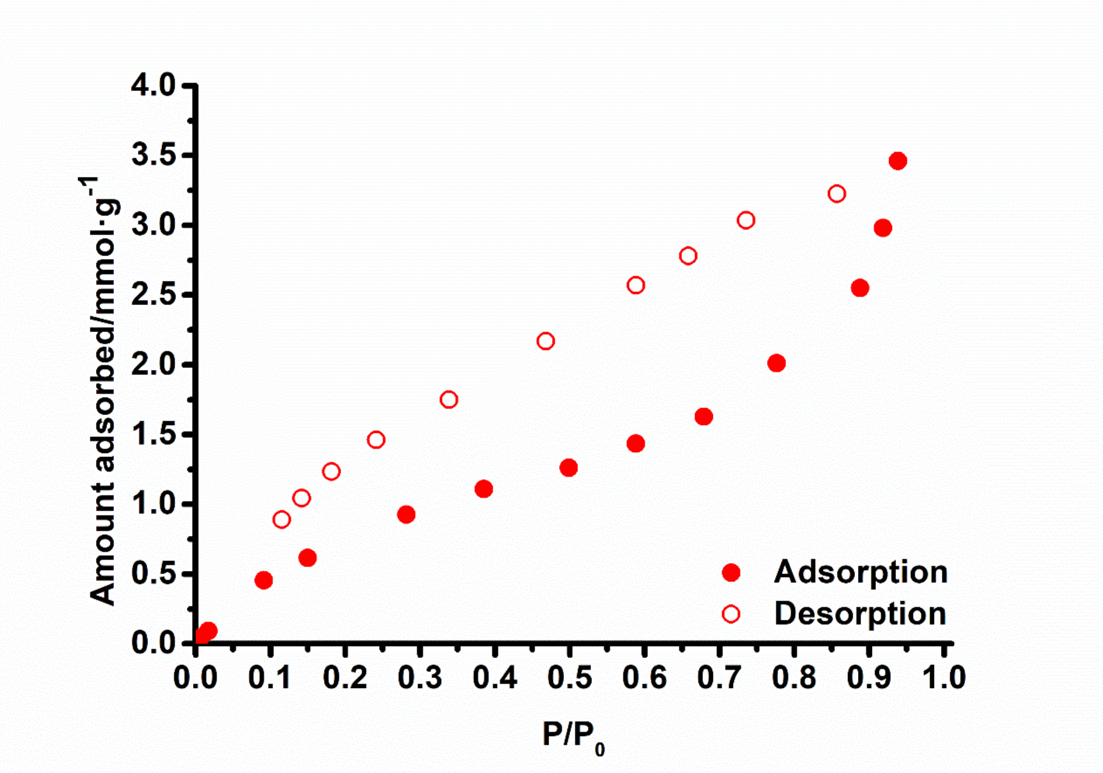

Supplementary Figure 26. Water vapor sorption of PCP-3,5-pdc at 298 K.

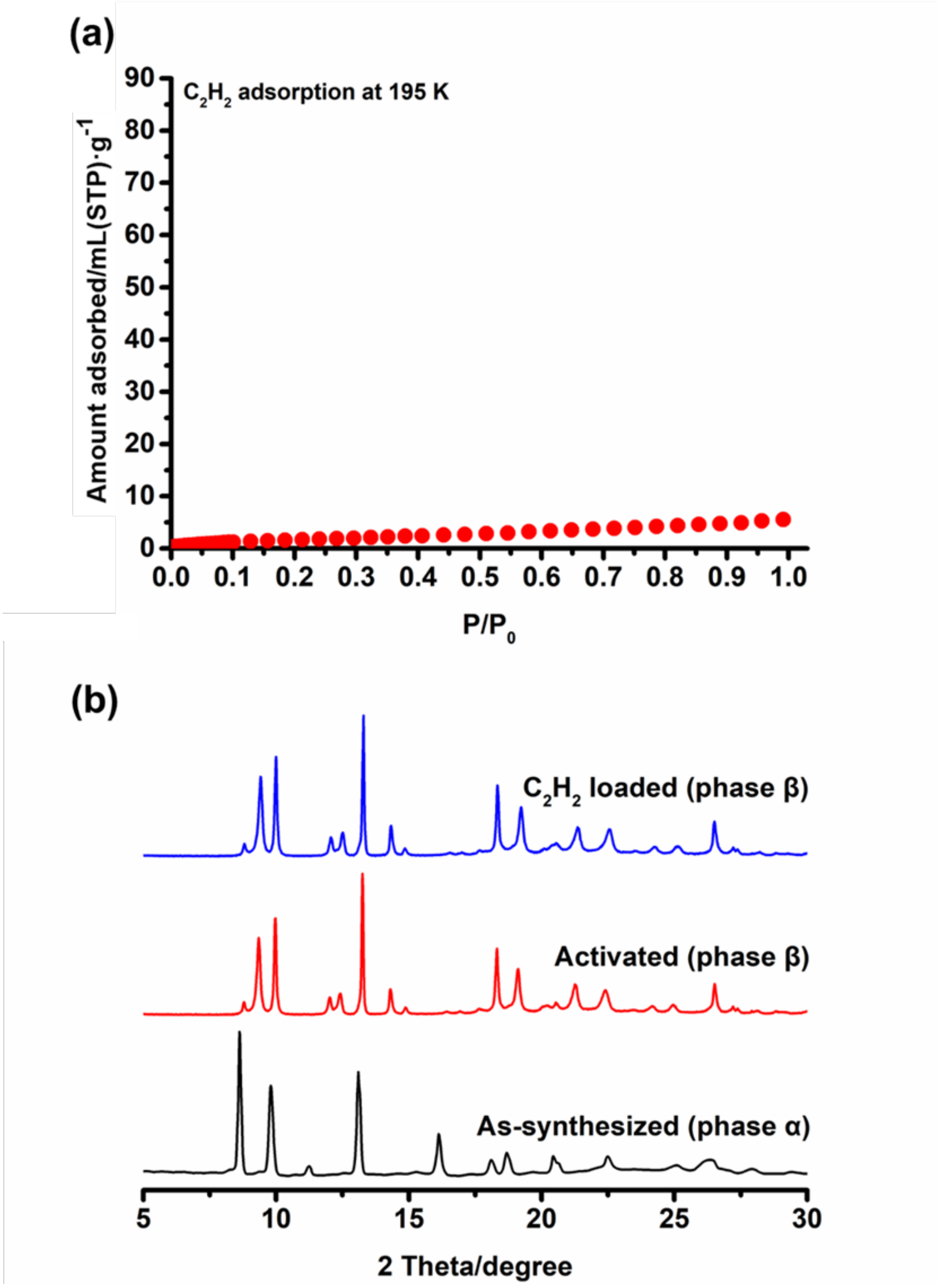

Supplementary Figure 27. In-situ synchrotron PXRD patterns of PCP-3,5-pdc accompanying C_2H_2 sorption at 195 K.

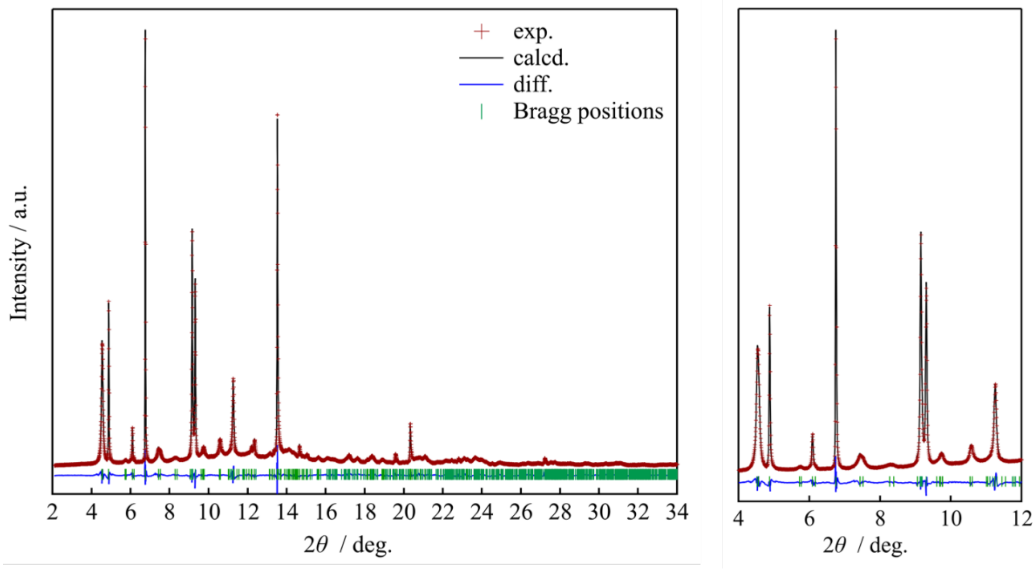

Supplementary Figure 28. Rietveld refinement of CO_2-loaded PCP-3,5-pdc at 195 K and 1 bar. R-value was converged to Rwp= 3.783, Rp= 2.711, S = 4.1301, R_B = 3.480 and R_F = 9.099.

Supplementary Table 5. Crystallographic data and structural refinement summary for CO_2 loading induced open phase structure of PCP-3,5-pdc.

	CO_2-loaded PCP-3,5-bdc
Chemical formula	C20.5CoN3O9
Formula weight	491.167
Crystal system	Triclinic
Space group	P-1
a/Å	10.1980(3)
b/Å	10.6478(4)
c/Å	10.6115(4)
α/Å	79.595(2)
β/°	72.340(4)
γ/°	73.295(4)
Cell volume / $Å^3$	1046.1
Z	4
Temperature/K	195
Wavelength/Å	0.799671(1)
2θ range/°	2.100 – 78.216
R_p	5.55%
R_{wp}	7.36%
R_{exp}	1.02%
Temperature/K	298
Wavelength/Å	0.799671(1)
2θ range/°	2.100 – 78.216
R_p	2.711%
R_{wp}	3.783%
S	4.1301%
R_B	3.480%
R_F	9.099%

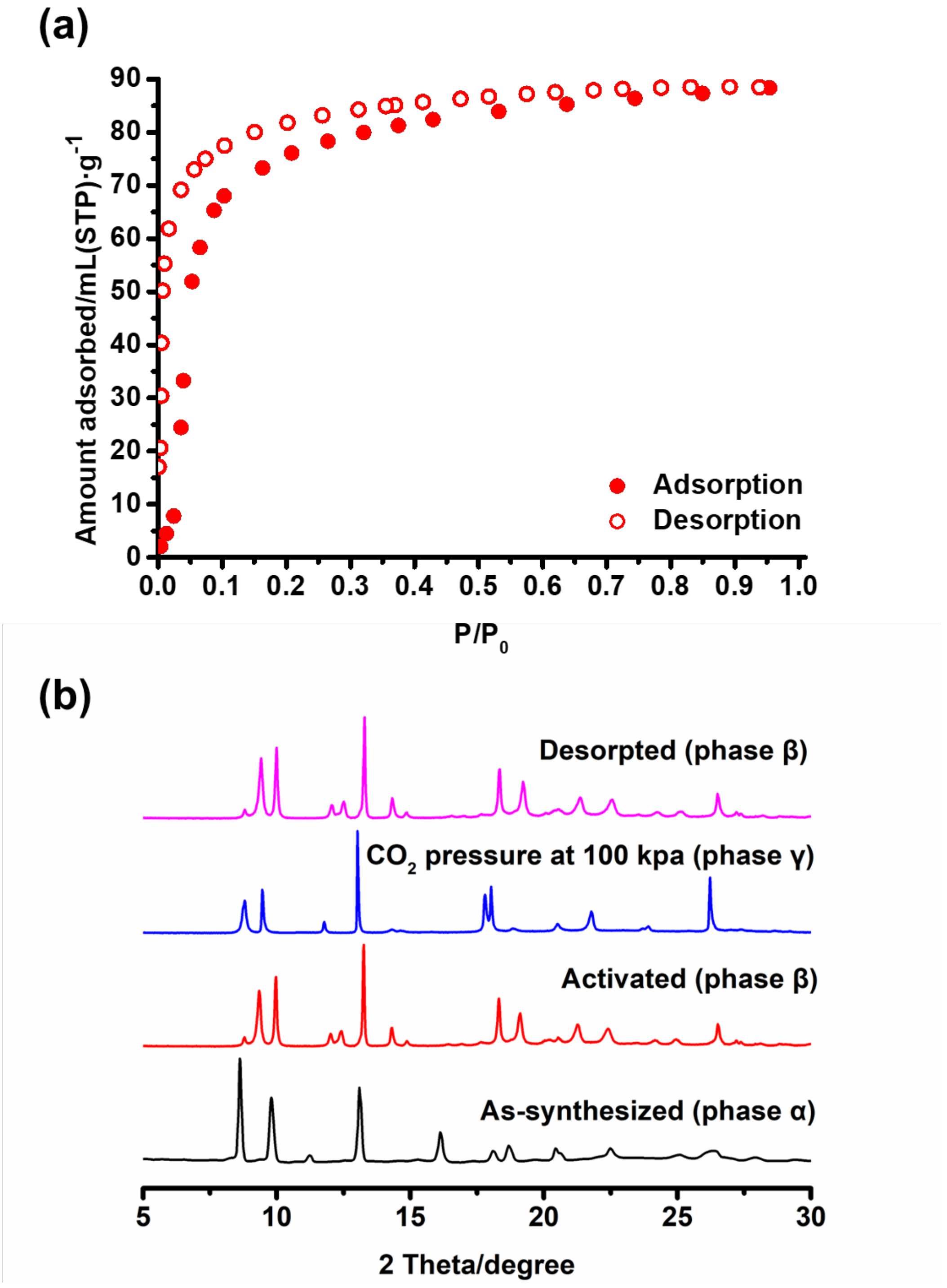

Supplementary Figure 29. In-situ synchrotron PXRD patterns of PCP-3,5-pdc accompanying CO_2 adsorption and desorption at 195 K.

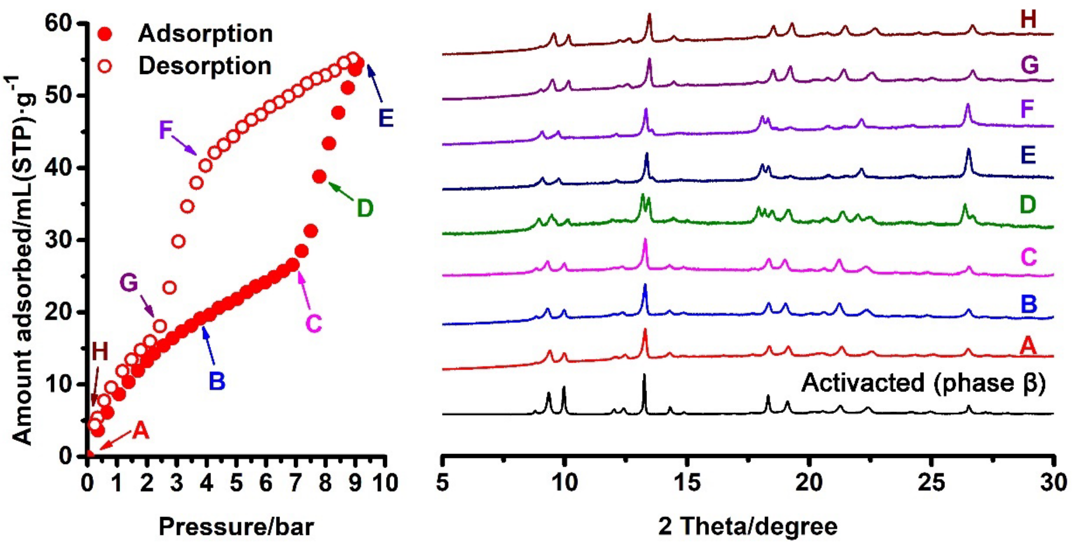

Supplementary Figure 30. In-situ PXRD patterns of PCP-3,5-pdc accompanying CO_2 sorption at 298 K up to 10 bar.

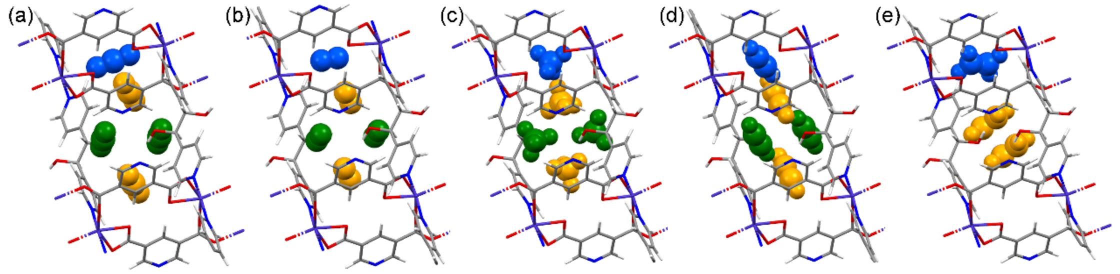

Supplementary Figure 31. Plausible adsorption positions of (a) CO_2, (b) N_2, (c) CH_4, (d) C_2H_2, and (e) C_2H_4 in PCP-3,5-pdc. On the basis of experimentally-determined CO_2 adsorption structures, these positions were grouped into two different sites, namely sites I (blue) and II (gold and green); site II has two possibilities which were named IIA (gold) and IIB (green), respectively. Due to the larger size of C_2H_4 than other gas species, site IIB was not located for C_2H_4 adsorption.

Supplementary Table 6. PBE-D3-calculated binding energies (BE, kcal mol^{-1}), interaction energies (E_{int}, kcal mol^{-1}) between gas molecule and framework, deformation energies (E_{def}, kcal mol^{-1}) of framework and volume changes (ΔV, $Å^3$) of framework upon adsorption of CO_2, N_2, CH_4, C_2H_2 and C_2H_4 into PCP-3,5-pdc at different adsorption sites.

	BE (kcal mol^{-1})			E_{int} (kcal mol^{-1})			E_{def} (kcal mol^{-1})			ΔV ($Å^3$)		
	I	IIA	IIB	I	IIA	IIB	I	IIA	IIB	I	IIA	IIB
CO_2	-8.6	-2.4	-1.0	-9.0	-8.6	-7.0	0.4	6.1	6.0	2	106	108
N_2	-6.1	0.1	1.1	-6.5	-5.2	-4.7	0.4	5.3	5.8	4	100	98
CH_4	-7.6	-1.2	-1.0	-7.9	-7.2	-7.1	0.3	5.9	6.0	3	106	118
C_2H_2	-9.9	-3.6	-3.4	-10.2	-10.3	-9.8	0.3	6.7	6.4	12	122	92
C_2H_4	-11.9	-2.7	n.d.	-12.2	-9.4	n.d.	0.3	6.7	n.d.	6	115	n.d.

For all gas molecules, the BE at site I is more negative than that at site II, suggesting that site I is better for the initial gas adsorption. This is because adsorption at site I induces little volume expansion of PCP-3,5-pdc framework, whereas adsorption at site II induces much larger volume expansion. Thus, the deformation energy (E_{def}) of framework upon gas adsorption is much larger at site II than at site I, leading to the smaller BE (less negative) at site II, despite the interaction energies (E_{int}) between gas molecule and framework are similar at site I and II.

Supplementary Table 7. PBE-D3-calculated binding energies (BE, kcal mol^{-1}), interaction energies ($E_{int,H-G}$ kcal mol^{-1}) between gas molecule and framework and that ($E_{int,G-G}$ kcal mol^{-1}) between gas molecules, deformation energies (E_{def}, kcal mol^{-1}) of framework for subsequent CO_2 adsorption into PCP-3,5-pdc at different loading.[a]

No. of CO_2 molecules per unit cell	BE (kcal mol^{-1})	$E_{int,H-G}$ (kcal mol^{-1})	$E_{int,G-G}$ (kcal mol^{-1})	E_{def} (kcal mol^{-1})
2	–3.0	–8.0	–0.1	5.1
3	–6.7	–9.7	–0.6	3.6
3.5[b]	–6.7	–9.8	–0.8	3.9
4	–5.6	–8.3	–1.0	3.7

[a] The most stable adsorption structure at different CO_2 loading was used to evaluate BE.

[b] Two unit cells were used in the calculation by removing one of the two CO_2 molecules adsorbed at site IIb.

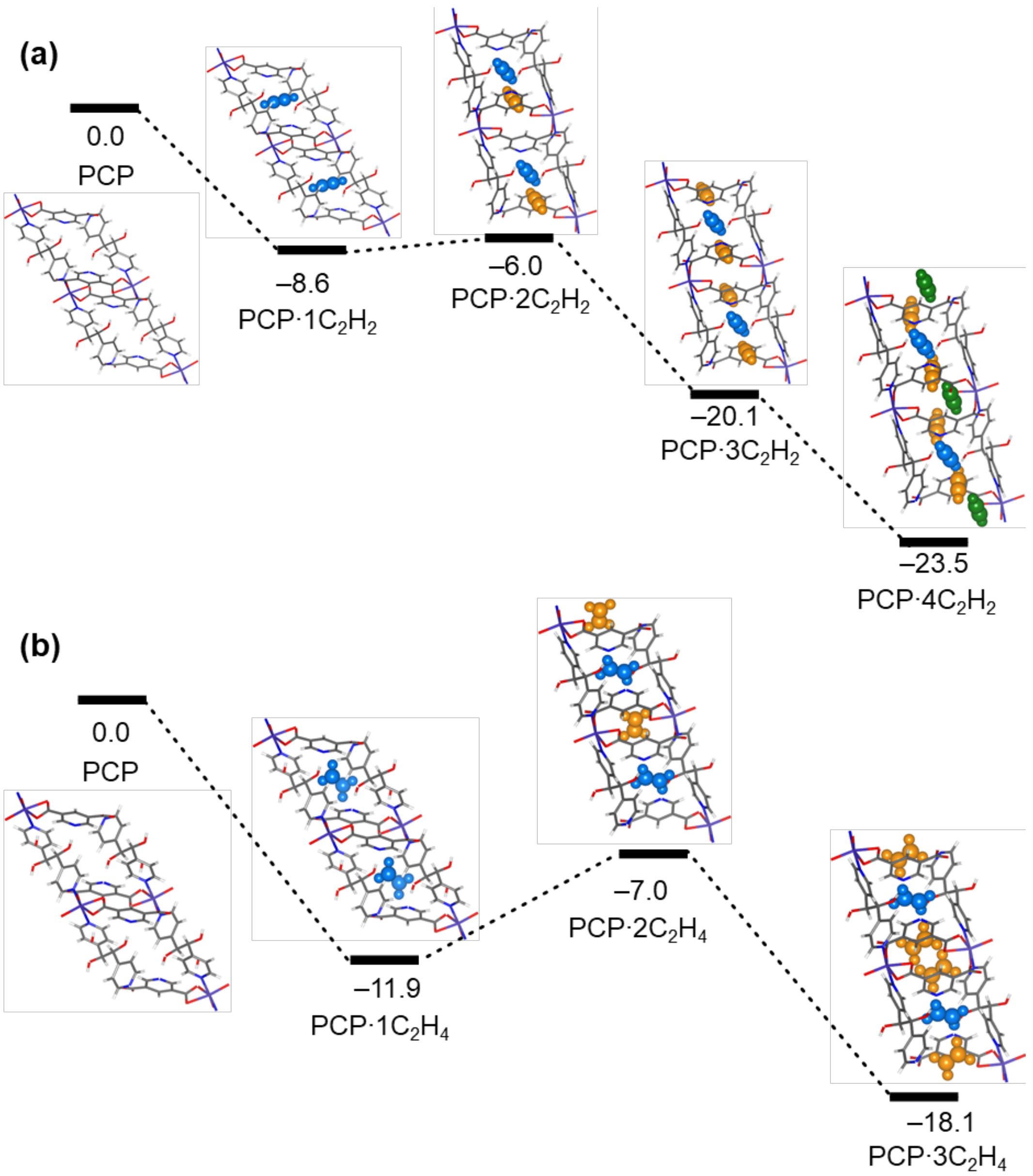

Supplementary Figure 32. Energy diagrams for (a) C_2H_2 and (b) C_2H_4 adsorptions into PCP-3,5-pdc. Energies are given in kcal mol^{-1}.

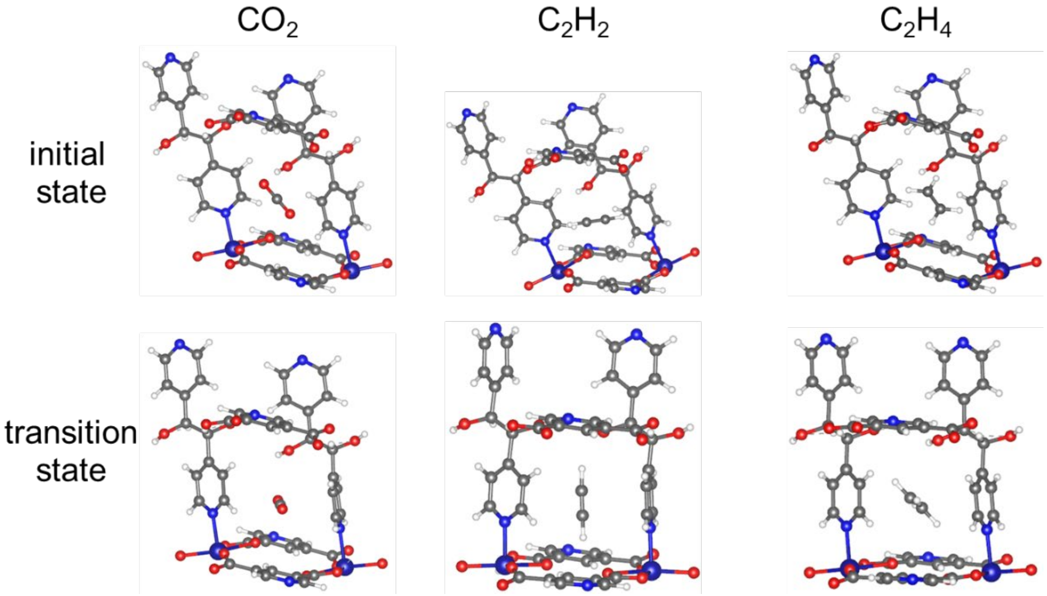

Supplementary Figure 33. Optimized structures of initial and transition states for diffusion of CO_2, C_2H_2, and C_2H_4 molecules through the narrow-corrugated channel. Gas molecules are located at site I at the initial state because that site I is the best for initial gas adsorption for all these gas species.

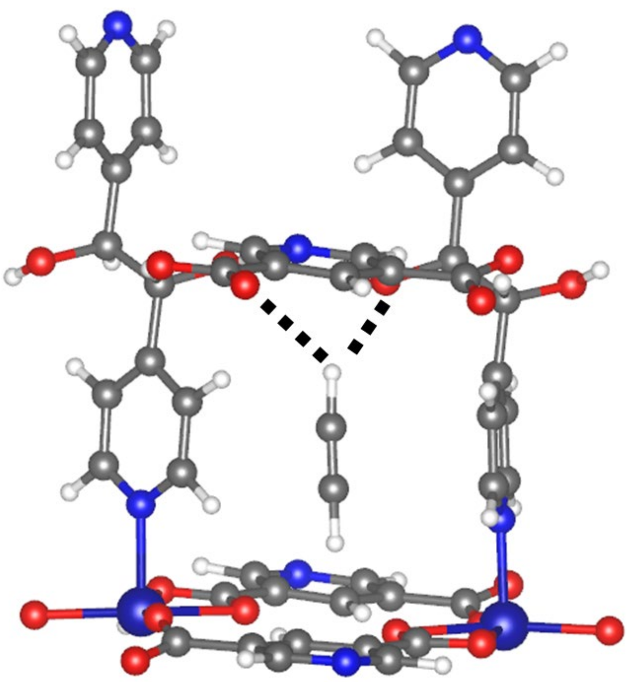

Supplementary Figure 34. The C-H···O interaction between C_2H_2 molecule and framework of PCP-3,5-bdc at initial state.

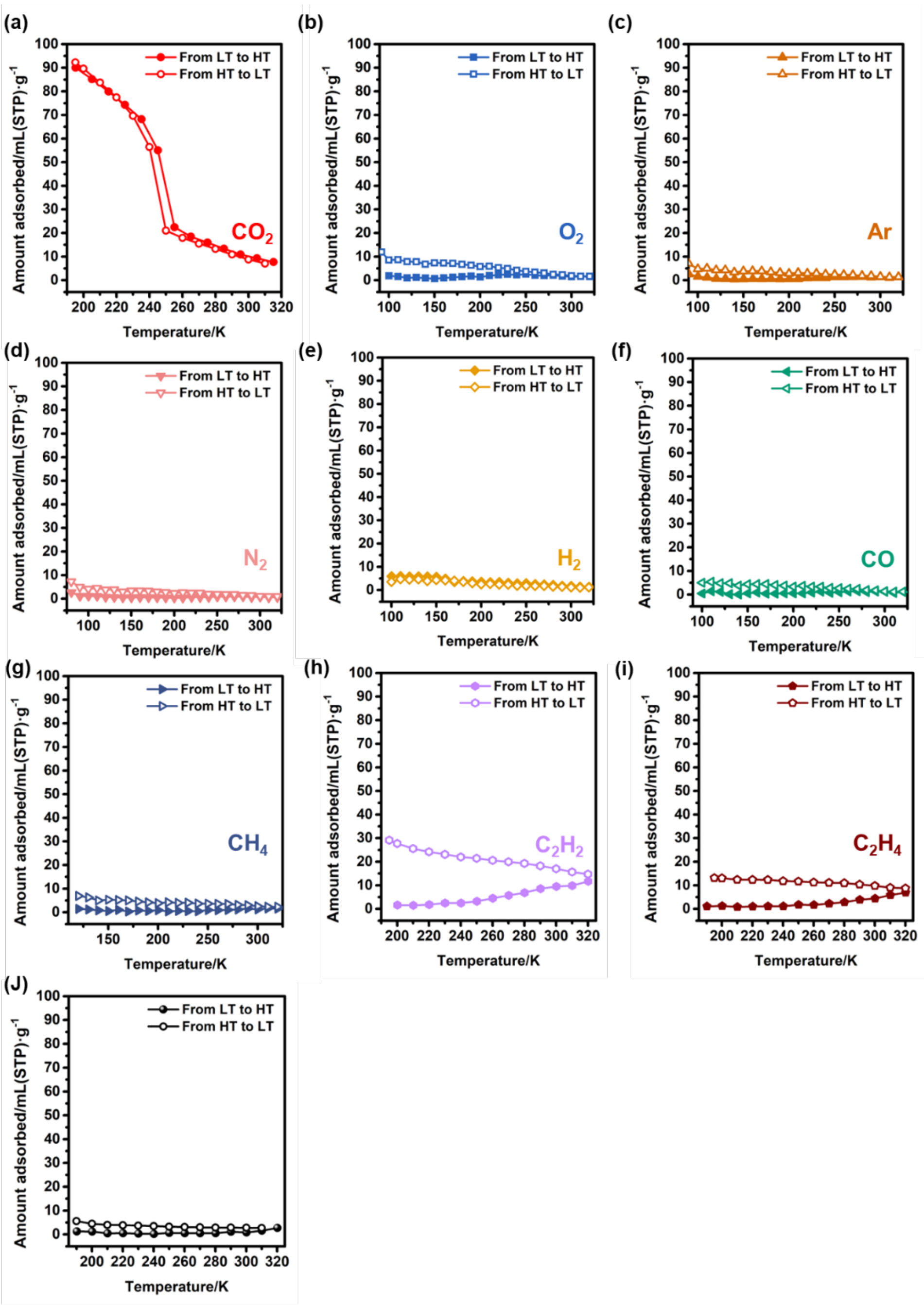

Supplementary Figure 35. Gas adsorption isobar measurements of (a) CO_2, (b) O_2, (c) Ar, (d) N_2, (e) H_2, (f) CO, (g) CH_4, (h) C_2H_2, (i) C_2H_4 and (j) C_2H_6 conducted from low temperature (LT) to high temperature (HT) and from HT to LT. When the C_2H_2 and C_2H_4 adsorption isobars were measured in the temperature direction of LT to HT, the adsorption amounts substantially increased. Such results suggesting the C_2H_2 and C_2H_4 can slightly enter the pore while increasing the temperature to facilitate their

diffusion. When the C_2H_2 and C_2H_4 adsorption isobars were measured in the temperature direction of HT to LT, the isobar curves isobar curves were obeyed the thermodynamic law. The adsorption amounts gradually increased with decreasing temperature. Such results further implied that the C_2H_2 and C_2H_4 adsorptions were influenced in the diffusion-limited channel. At low temperature, the diffusion of C_2H_2 and C_2H_4 was initially impeded and gradually boosted as increasing the temperature, leading to a temperature-assisted adsorption. On the contrary, the diffusion is expected to be easier if the initial adsorption happens at high temperature. After adsorbing gases, the diffusion barrier is further decreased, leading to an ordinary adsorption behavior. These results importantly indicated that the regulated adsorbates diffusion was one of the key determining factors for the observed recognition behaviour of CO_2 over C_2H_2 and C_2H_4.

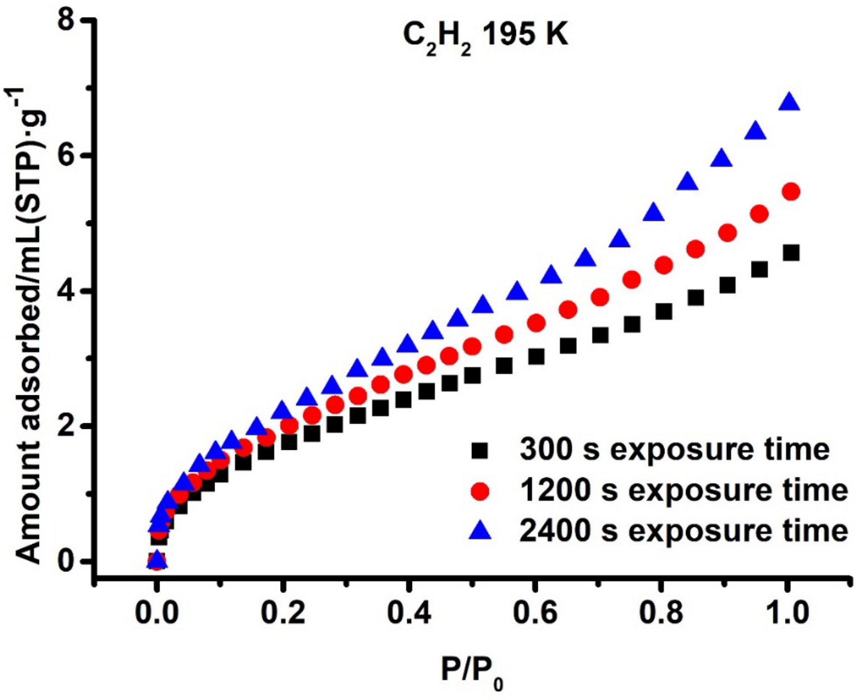

Supplementary Figure 36. C_2H_2 adsorption isotherms for PCP-3,5-pdc at 195 K using different exposure time. We measured the C_2H_2 adsorption isotherms at 200 K with the exposure time of each plot as 300 s, 1200 s and 2400 s, respectively. The adsorption amount slightly increased with the prolonged exposure time. However, no obvious gate-opening type sorption was observed. These results importantly indicated that the diffusion kinetics of adsorbates was one of the key determining factors for C_2H_2 adsorption.

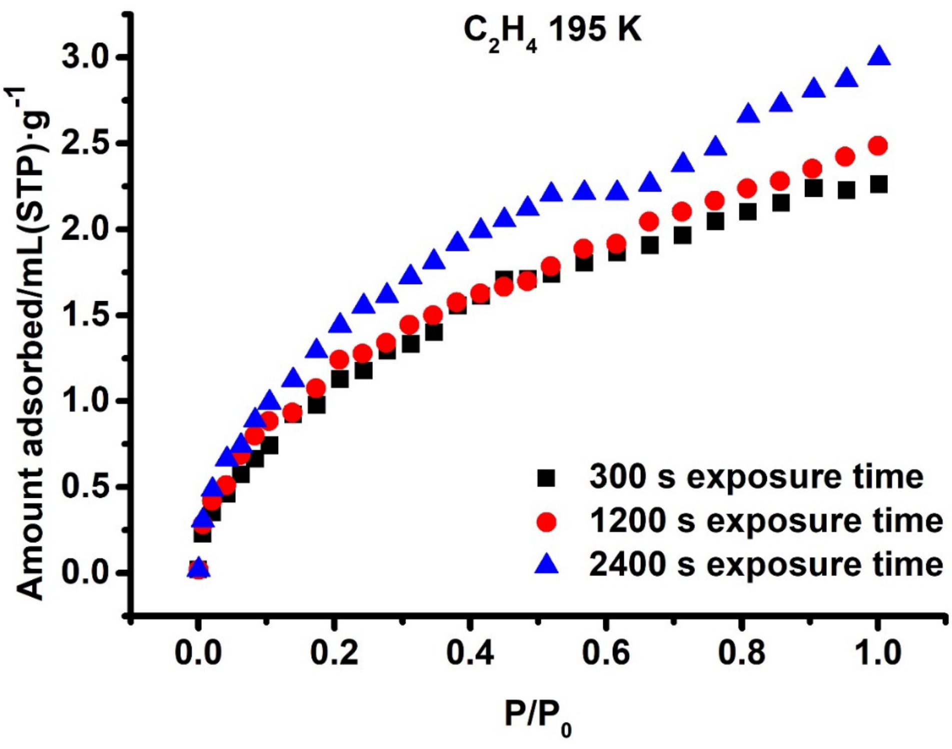

Supplementary Figure 37. C_2H_4 adsorption isotherms for PCP-3,5-pdc at 195 K using different exposure time. We measured the C_2H_4 adsorption isotherms at 200 K with the exposure time of each plot as 300 s, 1200 s and 2400 s, respectively. The adsorption amount slightly increased with the prolonged exposure time. However, no obvious gate-opening type sorption was observed. These results importantly indicated that the diffusion kinetics of adsorbates was one of the key determining factors for C_2H_4 adsorption.

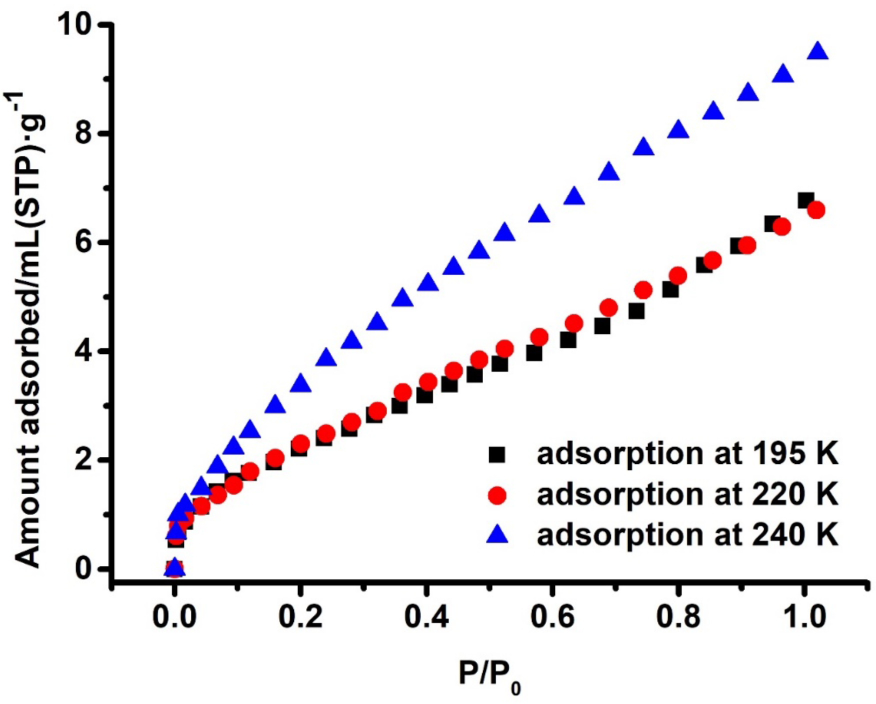

Supplementary Figure 38. C_2H_2 adsorption isotherms for PCP-3,5-pdc at 195 K, 220 K and 240 K using long exposure time (2400 s). We measured the C_2H_2 adsorption isotherms at 195 K, 220 K and 240 K with the exposure time of each plot as 2400 s. The adsorption amount slightly increased with the increment of measurement temperature. However, no obvious gate-opening type sorption was observed.

Supplementary Table 8. The CO_2 selective adsorption performance and main recognition mechanisms in representative PCPs.

PCP	Reported CO_2 selective adsorption performance	Main mechanisms
Qc-5-Cu-sql[6]	Selective CO_2 adsorption over CH_4 and N_2	Molecular sieving
Zr-FA[7]	Selective CO_2 adsorption over CH_4 and N_2	
$[Cu(bcppm)H_2O]$[8]	Selective CO_2 adsorption over N_2	
M-gallate (M = Mg, Co, Ni)[9]	Selective CO_2 adsorption over CH_4 and N_2	
ZU-610a[10]	Selective CO_2 adsorption over C_2H_2	Kinetic sieving
$[Cu_2(ndpa)]$[11]	Selective CO_2 adsorption over CH_4 and N_2	Interactions with open metal site
CPO-27-M (M = Mg, Co, Ni)[12]	Selective CO_2 adsorption over CH_4 and N_2	
$[Cu_2(OH)_2(bdim)]$[13]	Selective CO_2 adsorption over N_2	
MUF-16-M (M = Co, Mn, Ni)[14]	Selective CO_2 adsorption over CH4, C_2H_2, C_2H_4, C_2H_6, C_3H_6 and C_3H_8	Pore size and electrostatic potential complementary
$[Cu(tba)_2]$[15]	Selective CO_2 adsorption over CH_4, N_2, O_2, Ar, and H_2	host−guest C−H···O and guest−guest interactions
$[Mg_2(dobpdc)(eda)_{1.6}]$[16]	Direct air capture and CO_2 capture from flue gas	Interactions with appended amine
$[Mg_2(dobpdc)(mmen)_{1.6}]$[17]	Direct air capture and CO_2 capture from flue gas	
Mg2(dobpdc)(N,N′-bis(3-aminopropyl)-1,4-diaminobutane)[18]	Direct air capture and CO_2 capture from flue gas	
SIFSIX-3-Cu[19]	Direct air capture	Multiple weak interactions
SIFSIX-3-Zn[20]	Selective CO_2 adsorption over CH_4, N_2, and H_2	Multiple weak interactions
MAF-66[21]	Selective CO_2 adsorption over CH_4 and N_2	Interactions with multiple N
Co(bdp)[22]	Selective CO_2 adsorption over CH_4	The cooperation of framework flexibility and molecular sieving
$[Zn(5NO_2\text{-}ip)(dpe)]$[23]	Selective CO_2 adsorption over CH_4, C_2H_4 and C_2H_6	Framework flexibility
[Mn(bdc)(dpe)][24]	Selective CO_2 adsorption over C_2H_2	Framework flexibility
PCP-3,5-pdc (this work)	Selective CO_2 adsorption over N_2, CH_4, CO, O_2, H_2, Ar, C_2H_2, C_2H_4 and C_2H_6	The cooperation of pore stereochemical shape, location of binding sites, and framework flexibility

Supplementary References

1. Shimomura, S. et al. Selective sorption of oxygen and nitric oxide by an electron-donating flexible porous coordination polymer. *Nat. Chem.* **2**, 633-637 (2010).
2. Reid, C.R. & Thomas, K.M. Adsorption of Gases on a Carbon Molecular Sieve Used for Air Separation: Linear Adsorptives as Probes for Kinetic Selectivity. *Langmuir.* **15**, 3206-3218 (1999).
3. Bae, Y.S. & Lee, C.H. Sorption kinetics of eight gases on a carbon molecular sieve at elevated pressure. *Carbon.* **43**, 95-107 (2005).
4. Golden, T.C. & Sircar, S. Gas Adsorption on Silicalite. *J. Colloid Inter. Sci.* **162**, 182-188 (1994).
5. Vervoorts, P. et al. Coordinated Water as New Binding Sites for the Separation of Light Hydrocarbons in Metal–Organic Frameworks with Open Metal Sites. *ACS Appl. Mater. Inter.* **12**, 9448-9456 (2020).
6. Chen, K.-J. et al. Tuning Pore Size in Square-Lattice Coordination Networks for Size-Selective Sieving of CO2. *Angew. Chem. Int. Ed.* **55**, 10268-10272 (2016).
7. Shi, Y., Xie, Y., Alshahrani, T. & Chen, B. A zirconium-based microporous metal–organic framework for molecular sieving CO2 separation. *Cryst. Eng. Comm.* **25**, 1643-1647 (2023).
8. Bloch, W.M., Babarao, R., Hill, M.R., Doonan, C.J. & Sumby, C.J. Post-synthetic Structural Processing in a Metal–Organic Framework Material as a Mechanism for Exceptional CO2/N2 Selectivity. *J. Am. Chem. Soc.* **135**, 10441-10448 (2013).
9. Chen, F. et al. Carbon dioxide capture in gallate-based metal-organic frameworks. *Sep. Purif. Technol.* **292**, 121031 (2022).
10. Cui, J. et al. Kinetic-Sieving of Carbon Dioxide from Acetylene through a Novel Sulfonic Ultramicroporous Material. *Angew. Chem. Int. Ed.* **61**, e202208756 (2022).
11. Li, J.-R. et al. Porous materials with pre-designed single-molecule traps for CO2 selective adsorption. *Nat. Commun.* **4**, 1538 (2013).
12. Yu, D., Yazaydin, A.O., Lane, J.R., Dietzel, P.D.C. & Snurr, R.Q. A combined experimental and quantum chemical study of CO2 adsorption in the metal–organic framework CPO-27 with different metals. *Chem. Sci.* **4**, 3544-3556 (2013).
13. Zhou, D.-D. et al. A flexible porous Cu(ii) bis-imidazolate framework with ultrahigh concentration of active sites for efficient and recyclable CO2 capture. *Chem. Commun.* **49**, 11728-11730 (2013).
14. Qazvini, O.T., Babarao, R. & Telfer, S.G. Selective capture of carbon dioxide from hydrocarbons using a metal-organic framework. *Nat. Commun.* **12**, 197 (2021).
15. Du, M. et al. Divergent Kinetic and Thermodynamic Hydration of a Porous Cu(II) Coordination Polymer with Exclusive CO2 Sorption Selectivity. *J. Am. Chem. Soc.* **136**, 10906-10909 (2014).
16. Lee, W.R. et al. Diamine-functionalized metal–organic framework: exceptionally high CO2 capacities from ambient air and flue gas, ultrafast CO2 uptake rate, and adsorption mechanism. *Energ. Environ. Sci.* **7**, 744-751 (2014).
17. McDonald, T.M. et al. Capture of Carbon Dioxide from Air and Flue Gas in the Alkylamine-Appended Metal–Organic Framework mmen-Mg2(dobpdc). *J. Am. Chem. Soc.* **134**, 7056-7065 (2012).

18. Kim, E.J. et al. Cooperative carbon capture and steam regeneration with tetraamine-appended metal-organic frameworks. *Science.* **369**, 392-396 (2020).
19. Shekhah, O. et al. Made-to-order metal-organic frameworks for trace carbon dioxide removal and air capture. *Nat. Commun.* **5**, 4228 (2014).
20. Nugent, P. et al. Porous materials with optimal adsorption thermodynamics and kinetics for CO2 separation. *Nature.* **495**, 80-84 (2013).
21. Lin, R.-B., Chen, D., Lin, Y.-Y., Zhang, J.-P. & Chen, X.-M. A Zeolite-Like Zinc Triazolate Framework with High Gas Adsorption and Separation Performance. *Inorg. Chem.* **51**, 9950-9955 (2012).
22. Taylor, M.K. et al. Near-Perfect CO2/CH4 Selectivity Achieved through Reversible Guest Templating in the Flexible Metal–Organic Framework Co(bdp). *J. Am. Chem. Soc.* **140**, 10324-10331 (2018).
23. Horike, S. et al. Dense Coordination Network Capable of Selective CO2 Capture from C1 and C2 Hydrocarbons. *J. Am. Chem. Soc.* **134**, 9852-9855 (2012).
24. Foo, M.L. et al. An Adsorbate Discriminatory Gate Effect in a Flexible Porous Coordination Polymer for Selective Adsorption of CO2 over C2H2. *J. Am. Chem. Soc.* **138**, 3022-3030 (2016).
